# Fat and Happy: Profiling Mosquito Fat Body Lipid Storage and Composition Post-blood Meal

**DOI:** 10.3389/finsc.2021.693168

**Published:** 2021-06-16

**Authors:** Matthew Pinch, Soumi Mitra, Stacy D. Rodriguez, Yiyi Li, Yashoda Kandel, Barry Dungan, F. Omar Holguin, Geoffrey M. Attardo, Immo A. Hansen

**Affiliations:** ^1^Department of Biology, New Mexico State University, Las Cruces, NM, United States; ^2^Department of Computer Science, New Mexico State University, Las Cruces, NM, United States; ^3^Department of Plant and Environmental Sciences, New Mexico State University, Las Cruces, NM, United States; ^4^Department of Entomology and Nematology, University of California, Davis, Davis, CA, United States

**Keywords:** *Aedes aegypti*, lipidomics, lipid droplets, vitellogenesis, mass spectrometry

## Abstract

The fat body is considered the insect analog of vertebrate liver and fat tissue. In mosquitoes, a blood meal triggers a series of processes in the fat body that culminate in vitellogenesis, the process of yolk formation. Lipids are stored in the fat body in specialized organelles called lipid droplets that change in size depending on the nutritional and metabolic status of the insect. We surveyed lipid droplets in female *Aedes aegypti* fat body during a reproductive cycle using confocal microscopy and analyzed the dynamic changes in the fat body lipidome during this process using LC/MS. We found that lipid droplets underwent dynamic changes in volume after the mosquito took a blood meal. The lipid composition found in the fat body is quite complex with 117 distinct lipids that fall into 19 classes and sublcasses. Our results demonstrate that the lipid composition of the fat body is complex as most lipid classes underwent significant changes over the course of the vitellogenic cycle. This study lays the foundation for identifying unknown biochemical pathways active in the mosquito fat body, that are high-value targets for the development of novel mosquito control strategies.

## Introduction

Vitellogenesis in an autogenous mosquitoes depends on vertebrate blood. During oocyte development, female mosquitoes must produce large amounts of yolk proteins to provide the amino acid supply for the developing embryos. The necessary proteins for this process are provided by the vertebrate blood ingested when a female mosquito takes a blood meal. Proteins from the ingested blood meal are digested in the mid-gut and the amino acids along with lipids and carbohydrates are absorbed and transported to the fat body. The fat body is widely dispersed throughout the body of most insects. In the yellow fever mosquito, *Aedes aegypti*, a large portion of fat body tissue can be found lining the abdominal cuticle. In the fat body, digested amino acids are either metabolized or polymerized into yolk precursor proteins (YPPs) that are then exported to the developing oocytes in the ovaries (1).

In addition to its role in YPP production, the mosquito fat body is a central organ for energy homeostasis (2–5). Energy storage in the fat body occurs in specialized cells, called adipocytes or trophocytes. These large, polyploid cells store carbohydrate reserves as glycogen and amino acids as storage proteins. Lipid reserves are stored as triacylglycerides (TAGs) in large cytoplasmic droplets (6, 7). These lipid droplets are a conserved lipid storage mechanism found in all animals (6, 7). These droplets consist of a core of neutral glycerolipids surrounded by a phospholipid and protein monolayer (6, 7). Lipid droplets are formed in the smooth endoplasmic reticulum (ER), where they bud off the outer leaflet of the ER membrane (6, 7). The regulation of lipid droplet structure, and lipid storage are not well-understood, but their role as energy storage molecules makes them critical structures in the metabolism of lipids during vitellogenesis. In a previous study of lipid metabolism in *Ae. aegypti* fat body, the lipid droplet area, measured in confocal microscope images, decreased significantly between 6 h post blood meal (PBM) and 36 h PBM before rising back again by 72 h PBM (8).

Fat body lipid reserves are accumulated throughout the larval development of the mosquito and serve as a main energy source in adults. Studies of lipid metabolism in insects have shown that while TAGs are the most common lipid storage molecule, in many insects, diacylglycerides (DAGs) represent the most commonly transported lipid class (4, 9–14). However, in mosquitoes and other culicomorphs, TAGs are preferentially transported between tissues (15–17). Lipid transport through the hemolymph is mediated by lipophorin, a lipoprotein whose major lipid component is phospholipids (18, 19).

Earlier studies have shown that lipid levels in the fat body of female mosquitoes decrease significantly over the first 36 h post blood meal (PBM) (8, 20). This decrease corresponded with a decrease in lipid droplet size (8). In turn, another study found maximal lipid accumulation in the developing oocytes at 30 h PBM (21). These accumulated lipids comprise up to 30–40% of oocyte dry weight (22–24).

This study classifies changes in the variety of different lipid classes involved in *Ae. aegypti* fat body lipid metabolism and transport, as well as changes in lipid droplet structure in response to blood feeding. We present an in-depth, comprehensive time course profile of the *Ae. aegypti* fat body lipidome. This time course study includes analysis of changes in lipid classes, individual lipid molecules, and fatty acid chain length and saturation over the vitellogenic cycle. As mosquito populations continue to evolve resistance to existing classes of pesticides, it is imperative that we develop new means of controlling the spread of mosquito populations. The results of this study will deepen our understanding of *Ae. aegypti* fat body metabolism and provide a foundation for future studies designed to identify possible new targets for control of vitellogenesis and oocyte viability.

## Materials and Methods

### Mosquito Rearing

Lab-reared *Ae. aegypti* (Liverpool strain, NR-48921, BEI Resources) were used in this study. Larvae were reared in pans at low densities (<100 larvae/L) and fed *ad libitum* with “Special Kitty” cat food (Walmart, Bentonville, AR). Adults were housed in BugDorm-1 insect rearing cages (BioQuip, Compton, CA) at 26.5°C, 70% relative humidity and a 14:10 h light/dark cycle. Mosquitoes were fed a 20% sucrose solution *ad libitum*. Mosquitoes were separated for studies 7–10 days post emergence. Mosquitoes were starved for 16 h prior to administration of a blood meal. Mosquitoes were allowed to feed for 30 min. on defibrinated bovine blood (Hemostat Laboratories, Dixon, CA) warmed to 37°C.

### Fat Body Tissue Isolation

Female *Ae. aegypti* abdominal body walls were dissected in 1X modified *Aedes* physiological saline (mAPS) [sodium chloride [150 mM], sodium bicarbonate [23 mM], potassium chloride [4 mM], calcium chloride [2.5 mM], and magnesium chloride [0.8 mM]] (25) by removing the internal organs while retaining the cuticle and adhered fat body tissue (26). Samples were taken at the following time points: unfed (0 h post-blood meal), 30 min post-blood meal (PBM), 3 h PBM, 6 h PBM, 12 h PBM, 24 h PBM, 48 h PBM, 72 h PBM, and 96 h PBM.

### Nile Red Fat Body Lipid Droplet Staining Protocol

To quantify changes in lipid droplet size at different time points post blood meal, we stained dissected fat body tissue using the hydrophobic stain, Nile red (ab228553, Abcam, Cambridge, MA). Briefly, fat bodies from eight individual female *Ae. aegypti* per time point were dissected as described above. Dissected fat bodies were fixed in 4% paraformaldehyde for 30 min at room temperature, and then washed three times with 1X PBS. Fat bodies were stained with Nile red staining solution, prepared following manufacturer's instructions, for 30 min at 37°C, washed three times in 1X PBS, and counter stained with DAPI (NBD0015-1ML, Sigma, St. Louis, MO) diluted 1:1,000 in 1X PBS for 5 min at 37°C to mark nuclei. Nile red-stained lipid droplets were visualized on a Leica TCS-SP511 confocal microscope with 561 nm laser excitation. Emitted light was collected in a range from 565 to 650 nm. All images were taken at 63X magnification with a minimum resolution of 1,024 × 1,024 pixels. Because signal intensity was not going to be directly measured, image gain was adjusted to maximize signal/background ratio.

Lipid droplet area was calculated using ImageJ (27). Three images from each fat body at each time point (24 total images per time point) were used for analysis. Each image was threshold-adjusted using the default ImageJ settings to generate an eight-bit binary image. Binary images were analyzed using the Analyze Particles function to automatically generate measurements of lipid droplet areas. Analysis was performed three times to generate measurements of “small” (1–40 μm^2^), “medium” (41–100 μm^2^), and “large” (101-infinity μm^2^) lipid droplets. A circularity range of 0.1–1.00 was used to minimize measurement of background pixels. Measurements were evaluated against their corresponding source images, and area measurements of overlapping lipid droplets were discarded and manually re-annotated.

### Colorimetric Quantification of Glycerolipid Content

Glycerolipid content of the fat body at each time point listed above was determined using a colorimetric glycerol assay (Sigma, St. Louis, MO: Triglyceride Reagent T2449-10ML; Free Glycerol Reagent F6428-40ML; Glycerol Standard Solution G7793-5ML) using a protocol adapted from *Drosophila* metabolism studies (28). Three groups of 10 female *Ae. aegypti* were dissected as described above, and the fat bodies pooled to produce three biological replicates for each time point. The pooled fat bodies were homogenized with a hand-held homogenizer (#47747-370, VWR, Radnor, PA) in 200 μL of ice cold 1X PBST (phosphate buffered saline + 0.05% Tween-20). The homogenate was incubated at 70°C for 10 min to denature native proteins, and stored at −80°C.

To prepare the colorimetric assay, three 15 μL replicates of the following triolein equivalent glycerol standard dilutions were prepared: 1.0, 0.5, 0.25, 0.125, 0 mg/mL (blank). Standard aliquots were pipetted into individual wells of a clear-bottom 96-well plate and mixed with 15 μL of triglyceride reagent (TR). All biological replicates were briefly spun to pellet any remaining solid material, and samples were diluted to a 1:4 ratio in PBST. Three 15 μL technical replicates of each biological replicate were pipetted into individual wells of three 96-well plates and mixed with 15 μL of 1X PBST to allow for quantification of free glycerol. Another three 15 μL technical replicates of each biological replicate were pipetted into individual wells of the same three 96-well plates and mixed with 15 μL TR to quantify glycerol released from digestion of glycerolipids. All reactions were incubated at 37°C for 25 min. Hundred μL of free glycerol reagent was added to each reaction and the reactions were incubated at 37°C for 5 min. Absorbance at 540 nm for each plate was read using a BioTek Eon plate reader (BioTek, Winooski, VT). Free glycerol background values were subtracted from TR-treated values, and these adjusted values were used to calculate glycerolipid concentration using the triolein-equivalent standard curve (28). Final glycerolipid concentrations were calculated by dividing the concentration calculated from the standard curve by the sample volume (15 μL = 0.015 mL), and then multiplying by the dilution factor. All glycerolipid concentrations are reported as (mg lipid/mL sample).

### Fat Body Lipid Extraction for LC/MS Analysis

To understand the changes in fat body lipid composition after blood feeding, we extracted lipids from three pools of 25 fat bodies at each of the time points listed above using the Folch lipid extraction method (29). Fat bodies were dissected as described above. Pools of dissected fat bodies were homogenized with a VWR hand-held homogenizer in 2:1 (v/v) chloroform/methanol (600 μL) for 7 min to disrupt the abdominal cuticle and ensure homogenization of the associated fat body tissue. After homogenization, the volume of the solution was brought back up to 600 μL with the addition of fresh 2:1 chloroform/methanol, and the samples were mixed by orbital shaking (200 rpm) for 20 min at room temperature. Next, 120 μL of methanol was added to each sample, and the samples were centrifuged (10,000 rpm) for 5 min at room temperature to pellet the remaining tissue. The supernatant was collected in a fresh Eppendorf tube, and 240 μL of chloroform was added to re-establish the 2:1 chloroform:methanol solvent ratio. A volume of 223 μL of 0.9% sodium chloride in water was added to each sample and samples were briefly vortexed to mix the polar and non-polar phases. Phase separation was facilitated by centrifugation (2,400 rpm) for 10 min at room temperature. The upper polar phase was removed, and the lower non-polar phase was gently washed with 1:1 (v/v) methanol/water (400 μL) a total of three times to remove remaining polar solutes. The washed lipid-containing lower phase was stored at −80°C prior to LC/MS analysis.

### LC/MS/MS Analysis of Fat Body Lipidome

Samples were dried under nitrogen and re-suspended in 300 μL 2:1 chloroform/methanol spiked with 12.5 ppm 1,2-diheptadecanoyl-sn-glycero-3-phosphoethanolamine, hereafter referred to as 17:0 PE (Avanti Polar Lipids, Alabaster, Alabama), as an internal control. Lipid samples were stored at 4°C prior separation by liquid chromatography with an Acquity Ultra Performance Liquid Chromatographer (Waters, Manchester, UK). Lipid samples were injected into an Acquity UPLC CSH C_18_ 2.1 × 100 mm, 1.7 μm column (Waters, Manchester, UK) and separated in a two-solvent gradient, beginning with a ratio of 60% Solvent A (60% acetonitrile, 40% water, 10 mM ammonium formate) to 40% Solvent B (90% isopropanol, 10% acetonitrile, 10 mM ammonium formate) and ending with a solvent ratio of 1% Solvent A to 99% Solvent B (30). Separation was carried out at a column temperature of 55°C, and a solvent flow rate of 0.4 mL per min (30). Two technical replicates of each sample were used, and fresh methanol and 17:0 PE blanks were injected at the start of the run and between each time point.

Mass spectrometry was performed on a quadrupole time-of-flight (QTOF) mass spectrometer (QTOF Ultima, Waters, Manchester, UK) equipped with a lockspray™ electrospray ion source coupled to a Waters Acquity UPLC system (Waters, Manchester, UK). Mass spectra were collected in the positive electrospray ionization mode (ESI+). The nebulization gas was set to 650 L/h at a temperature of 450°C, the cone gas was set to 15 L/H and the source temperature was set to 110°C. A capillary voltage and cone voltage were set to 2,800 and 35 V, respectively. The Q-TOF Ultima MS acquisition rate was set to 1.0 s with a 0.1 s interscan delay. The scan range was from 200 to 1,500 m/z. Data was collected in continuum mode. A lockmass solution of 50 ppm raffinose (503.1612 m/z) in 50:50 water:methanol was delivered at 20 μL/min through an auxiliary pump and acquired every 10 s during the MS acquisition.

Data-independent analysis was performed in positive ionization mode with a desolvation temperature of 450°C. Ten μL of one replicate from each time point were mixed to produce a sample for MS2 data-dependent analysis. Waters chromatogram files were converted to ABF files using the Reifycs Analysis Base File Converter (Reifycs, Tokyo, Japan) and annotated using MS-DIAL ver 3.90 (31). Neutral lipids were detected as ammonium adducts, and phospholipids were detected primarily as proton adducts. Lipid identification was performed with a MS1 mass tolerance of 0.5 Da and MS2 Mass tolerance of 0.1 Da. An alignment file was generated using a 12 h PBM time point file with a retention time tolerance of 0.05 min and a mass tolerance of 0.2 Da, and the peak heights of the alignment data were normalized to the internal standard peak height. Unknown lipids were filtered out of the dataset. Any lipids with a retention time shorter than 1.5 min were removed as this time was too short for MS detection in each run. To generate a final lipidome for analysis, the remaining lipids were sorted by class, and manually re-annotated using mass to charge ratios (m/z) in the LipidMaps Structure Database Bulk Search with proton and ammonium adducts selected and a mass tolerance of 0.2. Any lipids that were automatically annotated as sodium adducts in MS-DIAL were removed from the dataset if there was no corresponding lipid match with a proton or ammonium adduct in LipidMaps.

### Statistical Analysis

Statistical analysis of lipid droplet area was performed with Kruskal-Wallis tests in R (32). As a part of lipid droplet area statistical analysis, fat body sample replicate effects were removed by linear modeling of fat body by time, to ensure that time was the only effect on lipid droplet size in our analysis. Glycerolipid assay data were tested for normality using a Shapiro-Wilk test using GraphPad Prism8 software (GraphPad Software, San Diego, CA). A one-way ANOVA was used to analyze glycerolipid assay data followed by a Tukey's HSD *post hoc* test to identify significant differences between time points using GraphPad Prism8 software (GraphPad Software, San Diego, CA). Sparse partial least squares discriminant analysis of the lipidome data was performed using Metaboanalyst (33). The R package, WGCNA (34) was used to generate a lipid dendrogram and modules of lipids with similar changes over the course of vitellogenesis. Statistical analysis of all lipid class time course data was performed with Kruskal-Wallis tests in R (32). The R package, ggplot2 (35) was used to generate plots of lipid classes with lipids that changed significantly over vitellogenesis. Spearman correlation coefficients were used to identify individual lipids that changed significantly over the course of vitellogenesis (32).

## Results

### Lipid Droplet Area

**Figure 1:**
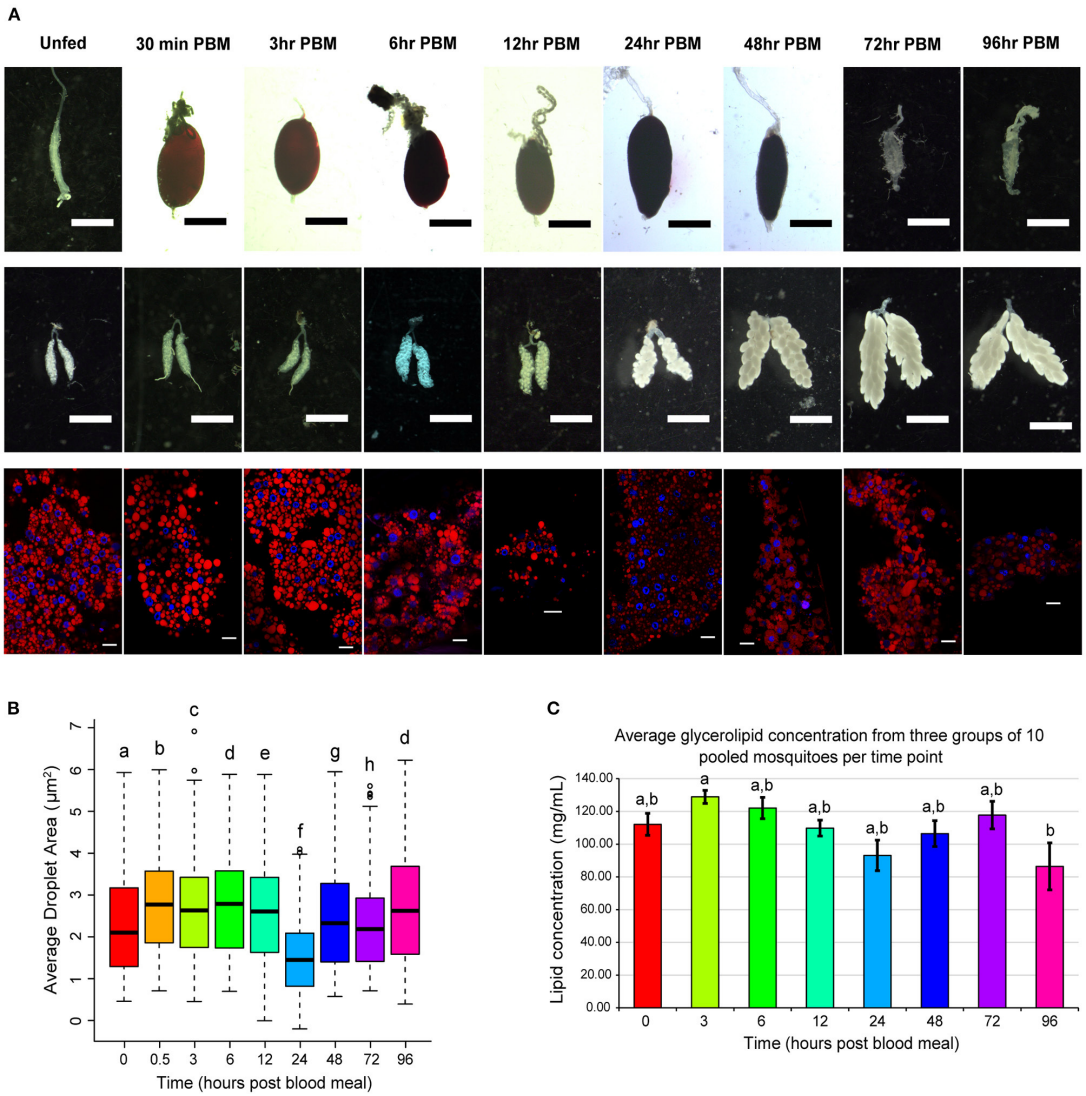
Changes in organ structure, fat body lipid droplets, and glycerolipid concentration over the course of the vitellogenic cycle. **(A)** Time course panel of Nile red stains/graphs of lipid droplet area along with corresponding ovary dissections to compare any differences in lipid droplet morphology to corresponding changes in oogenesis. Midgut and ovary scale bars are 1 mm; lipid droplet scale bars are 20 μm. Lipid droplet images were selected to be representative of the lipid droplet areas observed in this assay. **(B)** Average lipid droplet area. Boxplots illustrate 2nd−3rd interquartile range, and black lines inside boxes represent the median droplet area. Bars represent 1st and 4th quartiles, and circles represent outliers. Statistically significant changes were determined using a Kruskal-Wallis test, and letters represent statistically significant differences between time points (*p* < 0.05). **(C)** A colorimetric assay of glycerolipid concentration was performed on three pools of 10 female *Ae. aegypti* at nine time points across the vitellogenic cycle. After incubation with triglyceride reagent, free glycerol reagent was added, and absorbance at 540 nm was measured for all samples and background reactions. Background absorbances were subtracted from sample absorbances, and average lipid concentrations of each sample were calculated. Data is presented as average lipid concentration ± SEM. Statistically significant changes were determined using one-way ANOVA followed by a Tukey's HSD *post hoc* test, and letters represent statistically significant differences between time points (*p* < 0.05).

Because lipid droplets are the storage site of lipids, we chose to measure lipid droplet area to assay for changes in lipid storage and as an indicator of TAG content in adipocytes over the vitellogenic cycle. To place any observed changes more accurately in lipid droplet area within the larger context of physiological changes occurring in the vitellogenic cycle we show typical example images of mosquito midguts and ovaries taken at the same time point (Figure 1A upper two rows). We did not quantify changes in midgut or ovary morphology, but midgut size changed dramatically over the course of the vitellogenic cycle as the blood meal was digested (Figure 1A top row). Changes in ovary morphology were not apparent until 6 h PBM when the ovaries appeared to enlarge (Figure 1A middle row). At 24 h PBM, oocyte development was apparent, and eggs continued to enlarge through 72 h PBM (Figure 1A middle row). Nile red staining of the mosquito fat body revealed adipocytes with multiple large, usually spherical, or ovoid, lipid droplets. We observed changes in lipid droplet morphology (Figure 1A bottom row) over the course of the vitellogenic cycle.

We measured the area of lipid droplets from fat bodies of eight individual female *Ae. aegypti* mosquitoes from each timepoint to quantify structural changes in lipid droplets during the vitellogenic cycle (Figure 1B). Average lipid droplet area changed significantly across many of the time points in this study. The average lipid droplet area increased significantly from unfed to 0.5 h PBM and remained elevated through 12 h PBM. Lipid droplet area decreased significantly to the smallest point at 24 h PBM and increased back to similar levels as unfed by 48 h PBM.

### Lipid Quantification

In addition to analyzing changes in morphology of the fat body and lipid droplets, we investigated changes in lipid content of the fat body over the course of the vitellogenic cycle. To this end, we first performed a glycerolipid assay to measure changes in total glycerolipid content over the course of vitellogenesis (Figure 1C). Because the fat body produces large amounts of yolk precursor proteins during the vitellogenic cycle, we were unable to normalize our measured lipid concentrations to protein levels, as protein levels were also changing over the course of the experiment.

Average glycerolipid concentrations of three samples of 10 pooled fat bodies increased from 112.1 mg/mL in unfed fat bodies to 128.9 mg/mL at 3 h PBM (Figure 1C). We removed the 30 min PBM time point from our analysis of this assay, as the variability between sample groups was very high. From the peak concentration at 3 h PBM, glycerolipid concentrations steadily decreased to a significantly lower (*p* < 0.05) concentration of 86.43 mg/mL at 96 h PBM (Figure 1C). This decreasing trend was reversed at 48 h and 72 h PBM when an increase in average lipid concentration was observed (Figure 1C). These increases in lipid concentrations may be due to the recovery of lipid stores from the digested blood meal. The 96 h PBM time point had greater variability than any other time point, suggesting that the recovery of lipid storage in the fat body is a dynamic process that varies between mosquitoes.

### Lipidomics

#### General Description

Analysis of the total fat body lipidome across nine time points by UPLC/MS in positive ionization mode using low peak identification threshold parameters to capture a comprehensive set of compounds yielded a total of 704 compounds, 254 of which had an associated identifier in the MS-DIAL (31) database. We compared the identified lipid compounds to our data-dependent MS2 sample (see methods for sample preparation) to identify the major lipid classes present in our samples. Of the lipids present in our samples, 217 were annotated as glycerolipids and glycerophospholipids. The remaining 37 compounds were identified as sphingolipids. Manual re-annotation of these lipids using LipidMaps generated a group of 117 lipids that are included in subsequent analyses, including five putative wax lipids that were likely extracted from cuticle adhered to the fat body organ. We identified two neutral glycerolipid classes: diacylglycerol (DAG), and triacylgycerol (TAG), in our samples. We also identified the following glycerophospholipid subclasses: phosphatidic acids (PA) and lysophosphatidic acids (LPA), phosphatidycholines (PC) and lysophosphatidylcholines (LPC), phosphatidylethanolamines (PE) and lysophosphatidylethanolamines (LPE), phosphatidylglycerols (PG) and lysophosphatidylglycerols (LPG), phosphatidylinositols (PI) and lysophosphatidylinositols (LPI), and one lysophosphatidylserine (LPS). Finally, we identified three sphingolipid subclasses: sphingosine (SPB), sphingomyelins (SM) and ceramides (Cer), one cardiolipin (CL) and one cholesterol ester (CE). A spreadsheet containing normalized lipidome data and manual re-annotations is included (Supplementary File 1).

**Figure 2:**
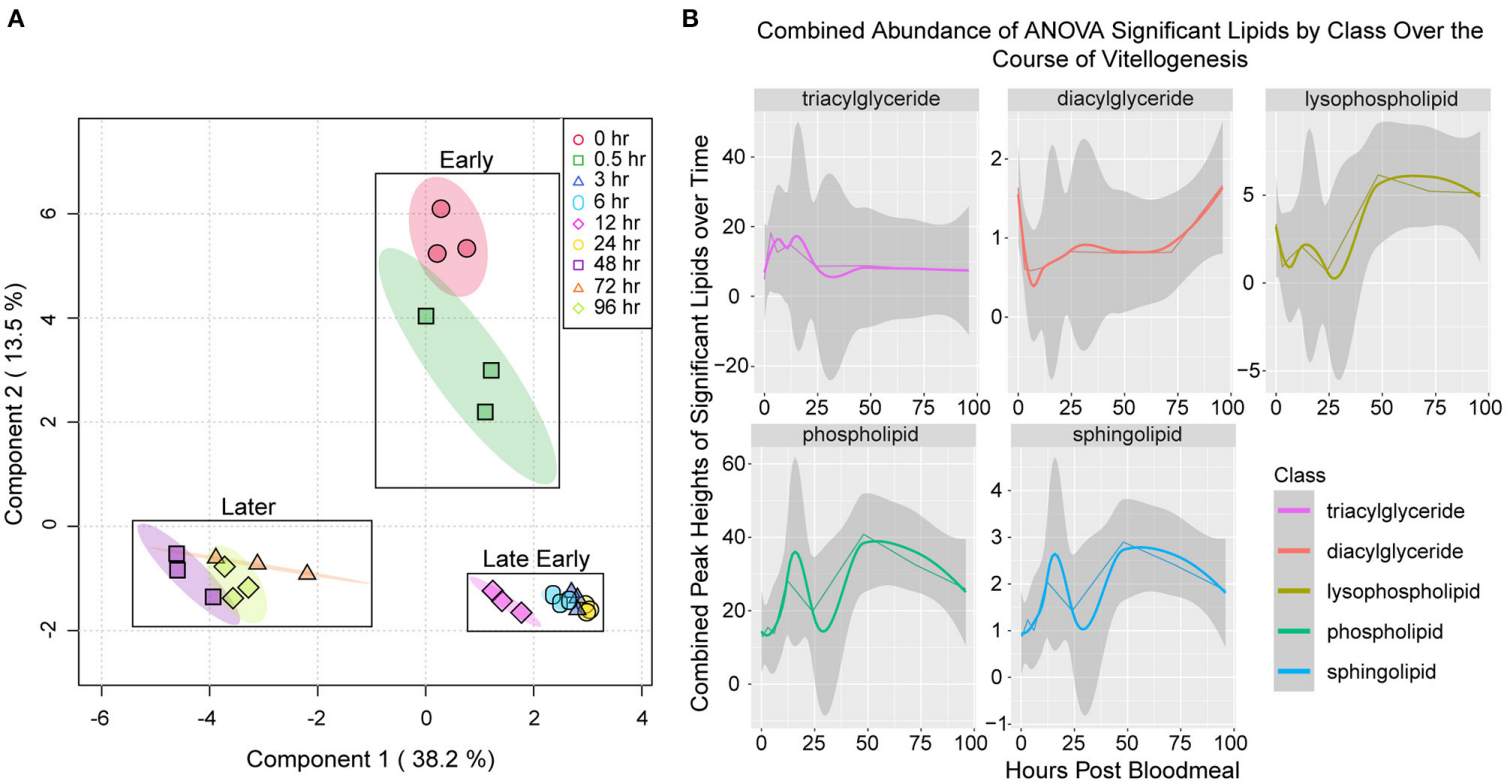
General patterns in variability of lipidomics data collected over the course of the vitellogenic cycle. **(A)** Sparse partial least squares analysis of fat body lipidomes sampled at nine time points during vitellogenesis. Colored circles represent biological replicates from each time point, while shaded ovals represent 95% confidence intervals. Boxes mark clusters of time points (early, late early, or later) during the vitellogenic cycle. **(B)** Fluctuations in lipid classes over the course of vitellogenesis. Values represent the sum of the peak heights of lipids within that class that were determined to change significantly over the time course by ANOVA using a cutoff of FDR adjusted *P* < 0.05. The gray bands around the lines represent the 95% confidence interval of the combined abundances of statistically significant lipids within that class of compounds. The confidence interval represents the potential variability derived from variance between the combined lipid values for each of the three biological replicates. The straight lines represent the actual observed abundance values, and smoothed lines represent local polynomial regression lines that were fitted to the observed data values using “loess” function within the stat_smooth function of ggplot2 (35).

We performed sparse partial least squares discriminant analysis on our lipidome data, and this revealed multiple clusters of related time points (Figure 2A). Early timepoints (unfed and 30 min PBM) clustered together, while late early time points (3 h PBM through 24 h PBM) clustered together. Later vitellogenic time points (48 h PBM through 96 h PBM) formed a third cluster. We also analyzed the levels of TAG, DAG, phospholipids, lysophospholipids, and sphingolipids that changed significantly over the course of the vitellogenic cycle (Figure 2B). TAG levels increased from unfed to three h PBM and fluctuated through 12 h PBM, where they decreased to their lowest point at 24 h PBM before beginning to recover at 48 h PBM (Figure 2B top left panel). We used the R package, WGCNA (34) to generate a dendrogram from our lipidome data (Supplementary File 2A). Many of the lipids clustered together into three modules each with a unique pattern of changes over the vitellogenic cycle. Neutral lipids clustered together into one module which decreased in abundance from 3 h PBM through 24 h PBM, while the other two modules contained different clusters of phospholipids and lysophospholipids with different abundance changes through vitellogenesis (Supplementary File 2B). Together, these data show a reduction in neutral lipid abundance through 24 h PBM, supporting previous reports of lipid transport from the fat body to oocytes occurring within the first 30–36 h PBM (21).

#### Differences Between Individual Lipid Classes Across Timepoints

**Figure 3:**
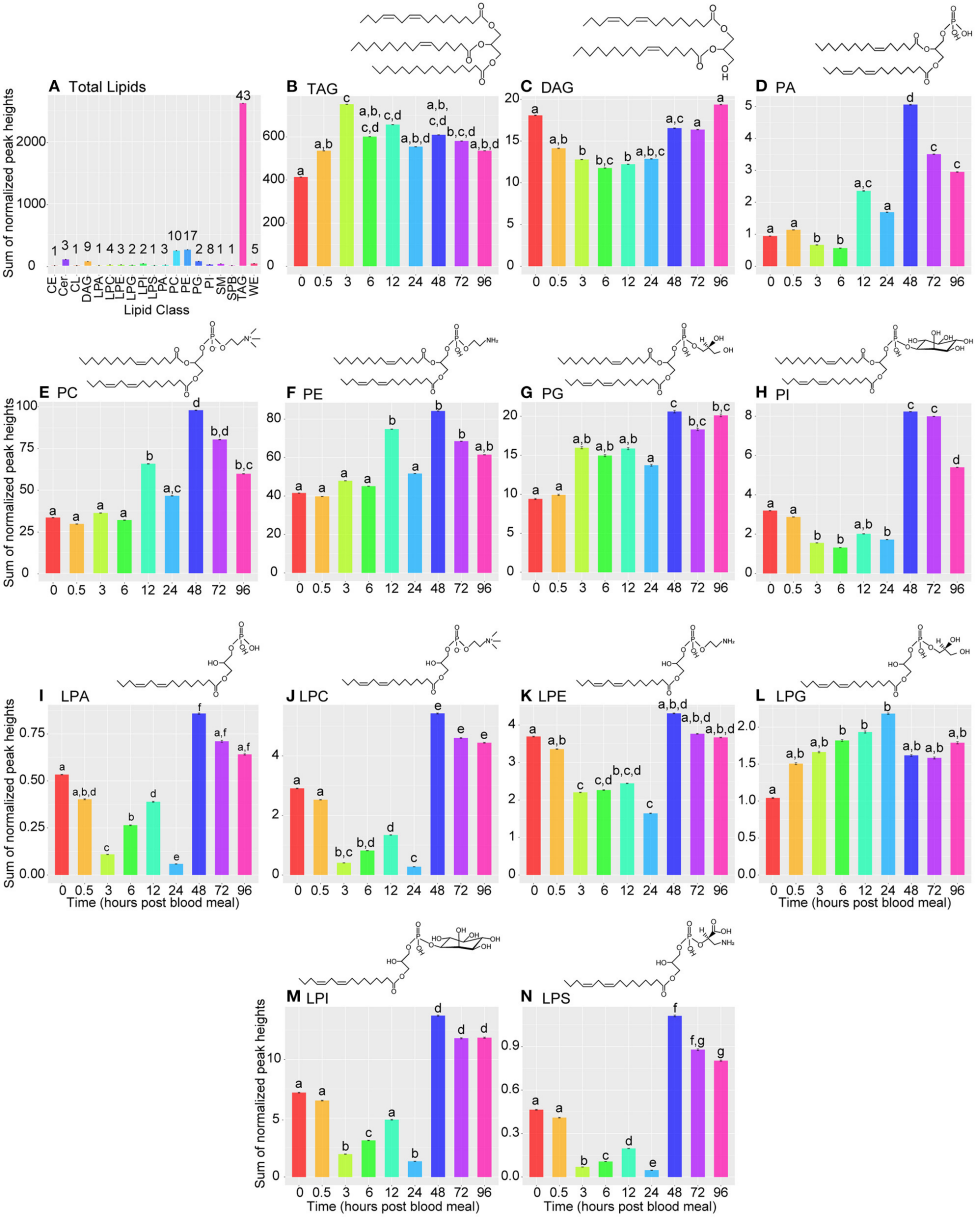
Bar graphs illustrating changes in lipid subclasses across time. **(A)** Bar graph illustrating lipid levels of 19 lipid subclasses measured by LC/MS at nine timepoints during the vitellogenic cycle. Lipid subclasses represented in this graph were identified in MS-DIAL and manually re-annotated using LipidMaps. CE, cholesteryl ester; Cer, ceramide; CL, cardiolipin; DAG, diacylglycerol; LPA, lysophosphatidic acid; LPC, lysophosphatidylcholine; LPE, lysophosphatidylethanolamine; LPG, lysophosphatidylglycerol; LPI, lysophosphatidylinositol; LPS, lysophosphatidylserine; PA, phosphatidic acid; PC, phosphatidylcholine; PE, phosphatidylethanolamine; PG, phosphatidylglycerol; PI, phosphatidylinositol; SM - sphingomyelin; SPB; sphinganine; TAG, triacylglycerol; WE, wax ester. Each remaining panel **(B–N)** represent changes in specified lipid subclasses over the 96-h vitellogenic cycle. Data are represented as means ± SEM. Statistically significant changes were determined using Kruskal-Wallis tests, and letters represent statistically significant differences between time points (*p* < 0.05). Note, the scale on the y-axis differs on each panel and represents the sum of normalized peak heights for all lipids within the given lipid class. Zero hour PBM time point represents unfed mosquitoes. Lipid drawings are example structures, and do not represent any specific lipid from our analysis.

TAG, PC, and PE species were the most abundant and diverse lipids among all time points (Figure 3A). The high abundance of TAGs is expected as they represent the major storage forms of lipids in animals. Similarly, relatively high amounts of PC and PE species is expected as PC and PE are a major lipid components of cell membranes, and abundant phospholipids in the phospholipid monolayer of lipid droplets (36, 37).

We next analyzed changes in each lipid class over the experimental time course. TAG levels increased significantly by 3 h PBM and decreased significantly again by 24 h PBM (Figure 3B). This general pattern in TAG levels matched the overall pattern in glycerolipid levels we observed in our colorimetric assay (Figure 1C), which gave us confidence that our lipidome data was representative of changes occurring in vitellogenesis. DAG levels decreased significantly by 3 h PBM and remained significantly lower than the unfed time point through 12 h PBM before beginning to recover at 24 h PBM (Figure 3C). All phospholipid subclasses have the same general pattern of lower levels through 24 h PBM, followed by a significant increase at 48 h PBM relative to unfed mosquitoes (Figures 3D–H). Lysophospholipids also followed this same pattern (Figures 3I–N), except for LPGs, which significantly increased by 6 h PBM before recovering to similar levels as unfed by 48 h PBM (Figure 3L). We also report changes in sphingolipid, CE, CL, and WE levels in Supplementary File 3.

We used Spearman correlation coefficients to determine individual lipids with significant changes in abundance across the vitellogenic cycle. We identified 33 lipids that changed significantly across our time course (Supplementary File 4). Two TAG species changed significantly over the vitellogenic cycle with one (TAG 42:0) significantly decreasing from unfed to 96 h PBM, and one (TAG 51:3) decreasing by 24 h PBM before significantly increasing by 48 h PBM and remaining elevated through 96 h PBM (Supplementary File 4). One DAG species (DG 44:3) decreased from unfed through 24 h PBM samples before significantly increasing at 48 h PBM (Supplementary File 4). Seven PC species changed significantly over the vitellogenic cycle, with all increasing by 48 h PBM and remaining elevated relative to unfed mosquitoes at 96 h PBM (Supplementary File 4). Eight PE species increased significantly in mosquitoes sampled from time points during the vitellogencic cycle (Supplementary File 4). Five PI species increased significantly in the later time points of the vitellogenic cycle (48 h PBM through 96 h PBM; Supplementary File 4). Two PA species (PA 36:8, PA 30:0) increased significantly by 12 h PBM and were elevated at 96 h PBM relative to unfed mosquitoes (Supplementary File 4). A single species of PG (PG 28:2) significantly increased over the course of the vitellogenic cycle (Supplementary File 4). Three lysophospholipids significantly changed in mosquitoes sampled at different time points during vitellogenesis. One (LPE 20:4) significantly decreased, while the other two (LPC 20:4 and LPI 22:1) significantly increased by 48 h PBM and remained elevated through 96 h PBM relative to unfed mosquito samples (Supplementary File 4). Three sphingolipids, one SM and two Cer, increased significantly over vitellogenesis (Supplementary File 4). The final lipid was a wax ester that likely was isolated from cuticle adhered to the fat body organ.

#### Lipidome Analysis for Carbon Chain Length and Double Bond Saturation

**Figure 4:**
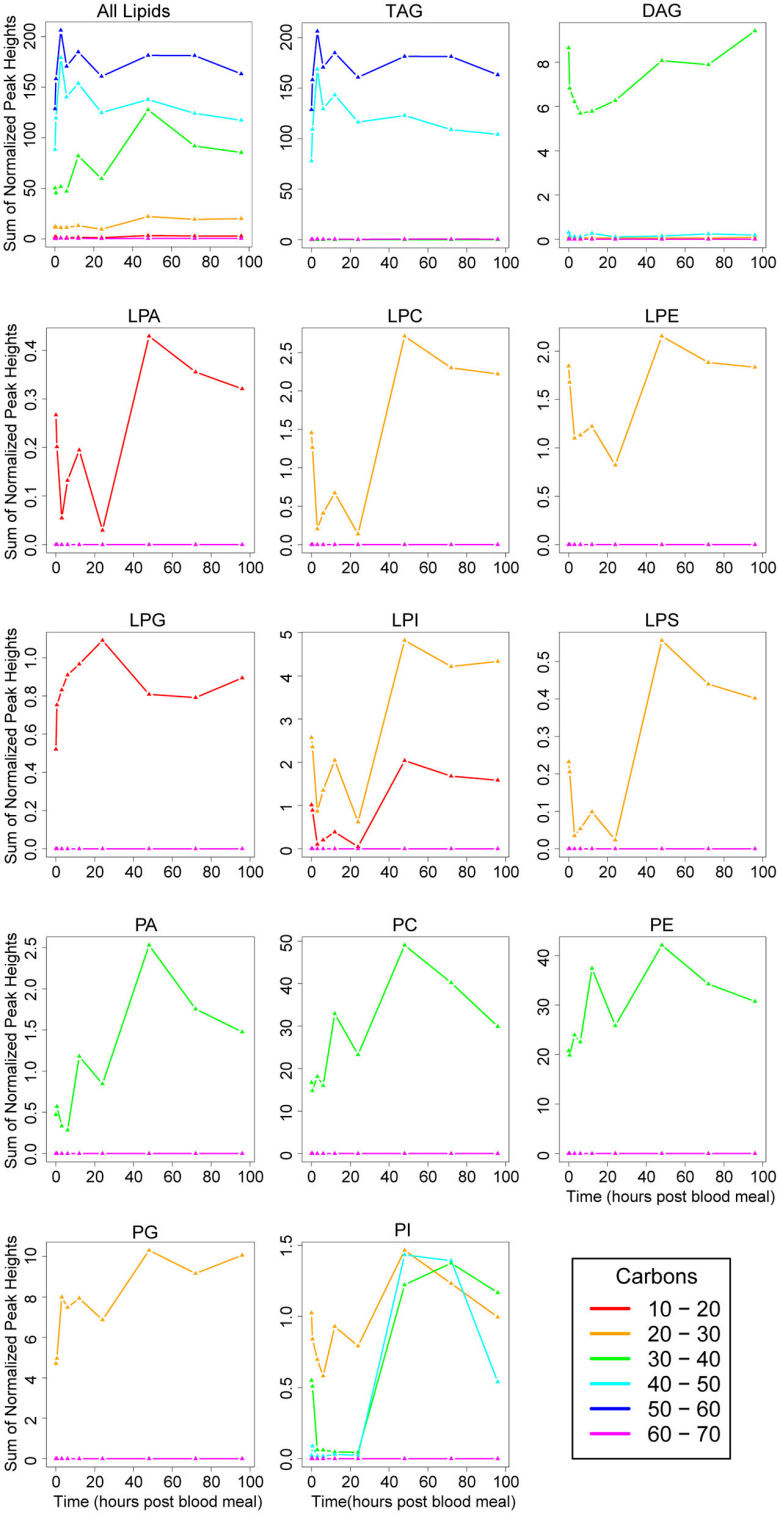
Changes in fatty acid chain length of lipid subclasses over the vitellogenic cycle. Changes in chain length across the whole lipidome are illustrated in the top left panel. Changes in each lipid subclass are shown in the remaining individual panels. Note, the scale on the y-axis differs on each panel, and represents the sum of normalized peak heights for all lipids within each chain length range (lines). 0 h PBM time point represents unfed mosquitoes. See Figure 3 for lipid subclass abbreviations.

We chose to survey carbon chain length and saturation of each glycerolipid subclass. We used total carbon number as a proxy for fatty acid chain length as each glycerolipid consists of a three-carbon backbone, so changes in carbon number represent changes in fatty acid chain length. The majority of molecules contained between 40 and 60 total carbons in their chains (Figure 4), likely because of the large amount of TAGs. Of the nine DAGs detected in our dataset (Figure 3A), five had 30–40 total carbons in their chains (Supplementary File 1). These represent the most abundant DAG molecules, and we observed a decrease in 30–40 carbon DAGs through 6 h PBM followed by a recovery in DAG levels by 48 h PBM (Figure 4). We observed a spike in lipids with 30–40 total carbons in their chains at 12 h PBM followed by a large increase between 24 and 48 h PBM (Figure 4), which reflects the overall trend we observed in phospholipid levels (Figure 2B bottom right panel). This trend is likely driven by the most expressed phospholipid subclasses, PC and PE (Figures 3A, 4). All lysophospholipids had chains containing between 10 and 30 carbons, with the lowest being 15 carbons (LPA 15:0), and the greatest being 22 carbons (LPI 22:1, LPS 22:4) (Supplementary File 1). We did observe some differences in fatty acid chain lengths in different nonpolar lipid classes (Figure 4). While all PA, PC, and PE lipids contained 30–40 total carbons in their chains, the two PG species we detected were shorter, containing 25 and 28 total carbons, and PIs had a greater variability in chain length than the other phospholipid subclasses (Figure 4, Supplementary File 1). Interestingly, a single PI with 26 total carbons in its chains (PI 26:0) was the most abundant PI through 24 h PBM, but by 48 h PBM, PIs with larger chains (30–50 total carbons) spiked to similar levels PI 26:0 (Figure 4). This spike in lipids with longer chains only at 48 h PBM is different than the pattern of phospholipid levels in the other subclasses, and even in PI 26:0 (Figure 4). As with PIs, LPIs were the only lysophospholipid class with variability in the range of carbons in their chains (Figure 4).

**Figure 5:**
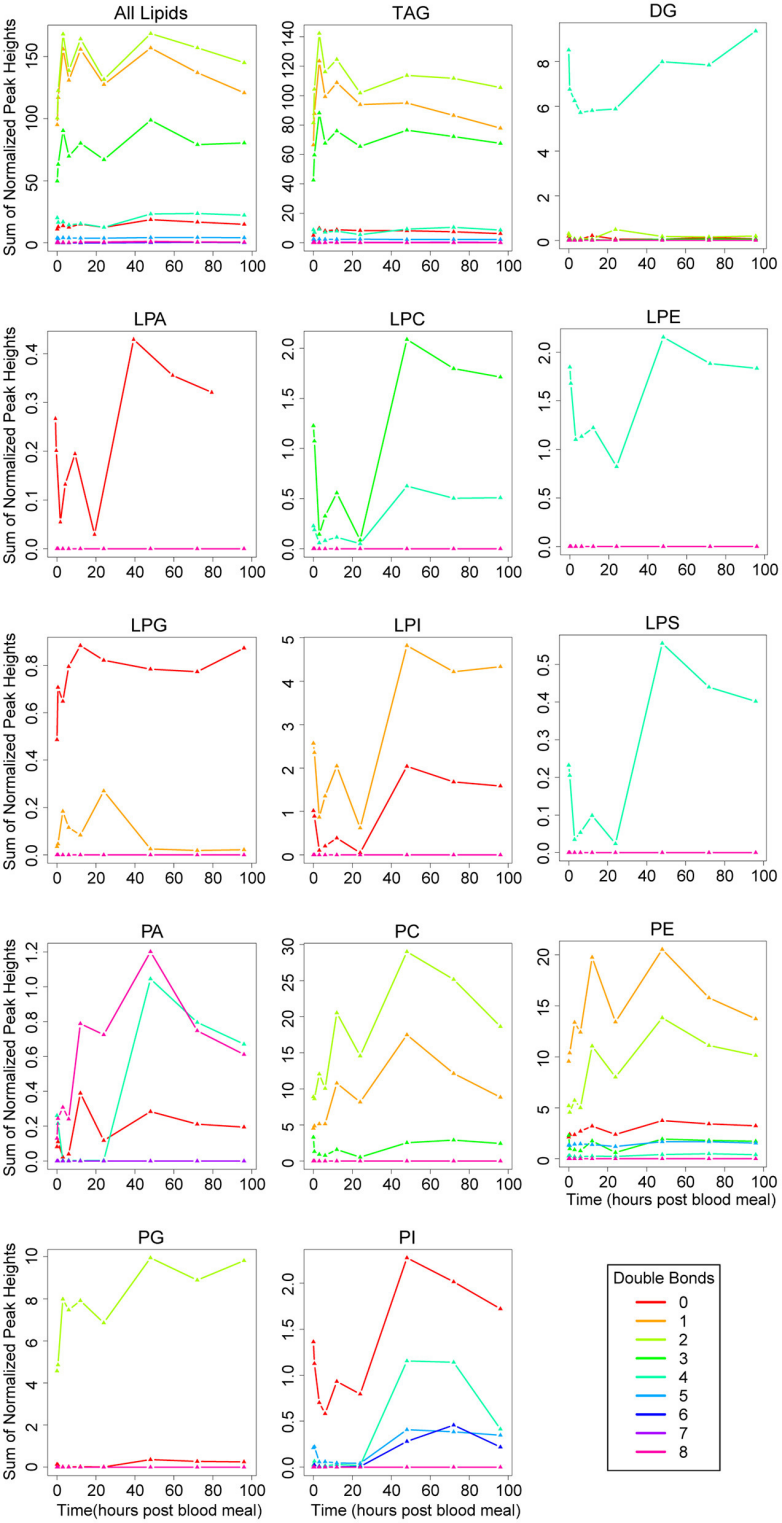
Changes in fatty acid saturation of lipid subclasses over the vitellogenic cycle. Changes in saturation across the whole lipidome are illustrated in the top left panel. Changes in each lipid subclass are shown in the remaining individual panels. Note, the scale on the y-axis differs on each panel, and represents the sum of normalized peak heights for all lipids within each saturation range (lines). 0 h PBM time point represents unfed mosquitoes. See Figure 3 for lipid subclass abbreviations.

Like the trends in fatty acid chain length, saturation trends observed across all lipids were very similar to the trends observed in TAGs (Figure 5). Overall, more than half of TAGs contained between one and three double bonds, and these were the most abundant TAG species (Figure 5, Supplementary File 1). Interestingly, more than half of DAGs including the two most abundant species in unfed mosquitoes (DAG 35:4, DAG 37:4) contained four double bonds, more than the number of most TAGs, even though they have one fewer fatty acid chain (Figure 5, Supplementary File 1). Lower numbers of double bonds and even unsaturated fatty acids seemed to be more common in TAGs than DAGs (Figure 5, Supplementary File 1). Phospholipids showed a greater variability in chain saturation across subclasses, with the two most interesting trends being spikes in lipid levels of four double bond-containing PA and PI molecules at 48 h PBM (Figure 5). Of interest, we observed several lysophospholipid subclasses with four double bonds in their single fatty acid chain (Figure 5). Two of these also have 20 carbon chains (LPC 20:4, LPE 20:4) (Figure 5, Supplementary File 1), so they may represent intermediates in arachidonic acid metabolism.

## Discussion

The metabolism of protein during the vitellogenic cycle of an autogenous mosquitoes, such as *Ae. aegypti* has been extensively studied (2, 3, 5, 38, 39). However, proteins represent only one macronutrient source that females must supply to developing oocytes. The metabolism of carbohydrates and lipids in the fat body of *Ae. aegypti* females during vitellogenesis is not as well-understood. To this end, we have performed a time course study of lipid droplet area and lipid content in the fat bodies of female *Ae. aegypti* (Liverpool) mosquitoes before blood feeding, and up to 96 h PBM. The results of this study demonstrate that lipid metabolism in the fat body of female *Ae. aegypti* during vitellogenesis is an extremely dynamic process.

We observed significant changes in lipid droplet area over the course of vitellogenesis with droplet areas reaching their lowest levels by 24 h PBM and recovering close to unfed by 48 h PBM. This pattern of decreasing lipid droplet area by 24 h PBM followed by an increase by 48 h PBM matches previous reports of lipid transport from the fat body to oocytes occurring within the first 30 h PBM (21), and of lipid droplet area decreasing over the first 36 h PBM (8). The observed increase in lipid droplet area from unfed to 0.5 h PBM which was sustained through the first 12 h PBM may be due to fusion of multiple lipid droplets as the large blood meals in the midguts of the mosquitoes (Figure 1, top) distended their abdomens and compressed the fat body tissue. Because we did not stain with a membrane marker, we cannot conclude that fusion is responsible for the increase in lipid droplet area. Future assays in lipid droplet area will include membrane markers to allow for determination of the number of lipid droplets observed per adipocyte. Alternatively, the observed increase in lipid droplet area may be caused by influx of digested lipids from the blood meals and/or synthesis of novel lipids from metabolites in the blood meals.

The general trend in lipid levels over the course of our experiment was similar when measured by a colorimetric assay of neutral lipids, and by whole lipidomics (Supplementary File 5). This general similarity is likely explained by the large proportion of triglycerides stored in the fat body, which are detected by both the colorimetric assay and LC/MS. We did observe a larger increase in total lipid levels at 12 h PBM in our whole lipidome data compared to our colorimetric data, which is likely due to increases in many phospholipids at that time point (Figures 2B bottom left panel, Figure 3D–H). The differences we observed between these two assays, namely a less visible decreasing trend in TAG levels in lipidome data in comparison to glycerolipid assay data, have several possible explanations. First, the colorimetric assay lipase may degrade other lipids such as diglycerides to produce the free glycerol measured in this assay, thus affecting the measured concentrations when compared to total lipid levels measured by LC/MS. Second, while fat bodies were rinsed after dissection, it is possible that contamination from the blood meal may have led to the presence of bovine proteins and lipids in the homogenate used for the colorimetric assay. We suspect that this may have been the reason for the high variability we observed at the 30 min PBM time point data from our glycerolipid assay analysis.

We expected to see a stronger downward trend in non-polar lipid levels (DAGs and TAGs) through the 24 h PBM time point, as it has previously been reported that lipid transport between the fat body and ovaries is completed within the first 30 h PBM (21), and TAG levels are significantly decreased in the fat body by 36 h PBM (8, 20). We hypothesize that the fluctuations we observed in lipid levels may be due to effluxes of pre-existing lipid stores concomitant with influxes of lipids from the blood meal. This is supported by evidence that female mosquitoes use their existing lipid reserves to supply oocytes in their first vitellogenic cycle, and that they use lipids from their blood meal to replace their fat body lipid reserves (40, 41). Future lipidomics studies integrating lipid content of different larval instars with pre- and post-blood fed mosquitoes will be useful for determining how energy stored in lipids is utilized throughout the entire life cycle of an autogenous mosquitoes.

We observed an increase in glycerolipid concentrations (Figure 1C) and in TAG from the lipidomics data (Figure 2B top left panel, Figure 3B) from unfed to 3 h PBM. This provides further support for the conclusion stated above that lipids from the digested blood meal contribute to an overall increase in lipid levels in the fat body shortly after the blood meal and prior to lipid export. Other researchers have determined the TAG concentration in bovine blood to be ~0.3 g/L (42). Additional work on a related mosquito species, *Culex quinqefasciatus*, demonstrated that females of this species ingest ~5 μL of blood to fully engorge (43), so we can assume the average TAG content of a bovine blood meal in this species is ~1.5 μg. We acknowledge that there are caveats when applying this calculation to our data including possible differences in lipid content of the blood from the reported cattle compared to those used for our blood meals, and the difference in mosquito species. But this provides more evidence that we could expect to see an increase in TAG levels in fat body after blood meal due to the amount of triglyceride ingested.

Regarding the different lipid classes observed in our assay, TAGs were the most abundant class of lipids, which is unsurprising considering their central role as lipid storage molecules. We did observe significant changes in TAG molecules over the vitellogenic cycle however (Figures 2B top left panel, Figure 3B, Supplementary File 4). While many TAGs decreased by 24 h PBM (Figures 2B top left panel, Figure 3B, Supplementary File 2), some TAG molecules increased over the course of vitellogenesis (Supplementary Files 2, 4). Those TAGs that decreased over the course of the study may be endogenously stored lipids that were being shuttled to the developing oocytes, while those TAG species that increased may be newly ingested or newly synthesized TAGs. We were surprised at the relatively low abundance of DAGs (Figure 3A), as it is a non-polar lipid that can be stored in lipid droplets and is the most commonly reported form of lipid transported by lipophorin in insects (19). It is possible that the lack of DAGs may be because they are synthesized from TAGs and rapidly exported into the hemolymph, thus representing a small portion of the fat body lipid population at any given time. However, we think it more likely that the lack of DAGs observed in our lipidome data provides further evidence that *Ae. aegypti* lipophorin preferentially transports TAGs as has been previously reported (15–17). In this study, we chose to acquire the MS data in positive ionization mode to generate a comprehensive glycerolipid profile as they are important lipid forms for energy storage and transport. Positive ionization lacks the sensitivity when compared to negative ionization mode for free fatty acids and sterols, and thus we lack coverage of these lipid classes. Future studies will include MS acquisition in negative ionization mode to ensure accurate profiling of free fatty acids and sterols, as they are important metabolic intermediaries and signaling molecules.

Phosphatidylethanolamines and phosphatidylcholines were the second and third most diverse and abundant lipid subclasses observed, respectively (Figure 3A). This is likely because these two phospholipids make up a large portion of cell membranes (44), and PCs are also the dominant phospholipidsfound in the polar lipid monolayer surrounding lipid droplets (36, 37). It has previously been reported that up to 90% of the lipid content in bovine serum lipoproteins consists of cholesteryl esters and phospholipids (45), so the increase in fat body phospholipid content at 48 h PBM may be caused by absorption of phospholipids from the bovine blood meal. It is also possible that this increase in phospholipid levels at 48 h PBM is due to re-modeling of lipids to re-establish membrane and lipid droplet monolayer composition after the large flux of lipids into and out of the fat body during the first 30–36 h PBM. The observed fluctuations in lysophospholipids (Figure 2B top right panel, Figures 3I–N) may be due to changes in phospholipid metabolism, or they may be due to changes in signaling in fat body adipocytes, as lysophospholipids have been shown to play roles as signaling molecules (46, 47), and several contain arachidonic acid chains (Supplementary File 1).

We also observed several lipids containing at least one odd-chain fatty acid (Supplementary File 1). This is interesting because animals normally synthesize and store lipids with even numbered fatty acid chains. We offer two explanations for the number of odd-chain fatty acids we observed in the *Ae. aegypti* fat body lipidome. First, these fatty acids may be stored fatty acids that the mosquitoes ingested from microbes in the water during larval rearing, or from microflora in the sugar solution they were raised on as adults prior to blood feeding. Second, mosquitoes were fed on bovine blood, and it is thought that ruminants may have relatively large amounts of odd-chain fatty acids contributed by their ruminal microflora (48). Finally, metabolism of branched chain amino acids (BCAAs) has been shown to generate 3-carbon molecules that can be incorporated into lipids during new fatty acid biosynthesis in adipocyte cell culture (49). Hemoglobin contains many of these BCAAs, so it is possible that digested amino acids from hemoglobin in the blood meal were synthesized into odd-chain fatty acids and incorporated during *de novo* lipid synthesis in the fat bodies of mosquitoes post-blood meal.

Our results demonstrate that lipid metabolism and lipid droplet size are dynamic during vitellogenesis. The dynamic nature of lipid droplets is not well-understood but has been a subject of greater study in recent years. Along with their role as lipid storage structures, lipid droplets have been shown to store and interact with a variety of proteins (50), including histones and spliceosomal factors in *Drosophila* cells (51, 52). It is possible that some changes we observed in lipid droplet size in our experiment may be due at least in part to storage and release of proteins necessary for changes in gene expression and metabolism of adipocytes during vitellogenesis. There is evidence that during vitellogenesis in *Ae. aegypti*, lipophorin does not load and unload lipids extracellularly, but is synthesized and loaded with TAGs in adipocytes and then exported into the hemolymph before being imported with its lipid cargo into the cells of the oocytes (16, 53). Additionally, lipophorin levels increase over the first 48 h PBM before decreasing again (54), mirroring our evidence and previous reports of lipid levels reaching their lowest levels in the fat body by 36 h PBM (8, 20).

The results of this study provide a strong foundation to design future studies for the analysis of lipid metabolism in the fat body of *Ae. aegypti* females during vitellogenesis. Future studies should use GC/MS to identify and quantify specific fatty acid species from our lipidome. Additionally, changes in lipid levels and lipid droplet size can be combined with proteomic and gene expression studies of enzymes involved in fatty acid synthesis and elongation, glycerolipid metabolism, phospholipid metabolism, unsaturated fatty acid metabolism, ether lipid metabolism, arachidonic acid and linoleic acid metabolism, fatty acid degradation, lipid droplet lipase (TG-lipase), lipid droplet associated proteins (Lsd1 and Lsd2), lipophorin, and enzymes involved in branched chain amino acid metabolism to further understand how lipid droplets and lipid metabolism are regulated during vitellogenesis. To continue to broaden our understanding of fat body metabolism during vitellogenesis, additional time course studies to analyze changes in metabolites and proteins involved in metabolite transfer, processing, synthesis, and gene regulation should be performed. These studies, combined with our time course lipidome data will provide the foundation for functional studies such as hormone manipulations using fat body culture methods previously established by our lab (26), or perturbation of lipid droplet proteins to elucidate signaling and metabolic molecules controlling lipid metabolism, and loading and export of TAG-lipophorin that can be used as targets for novel insecticides.

In addition to determining direct targets for new insecticides, this lipidome data can be useful for studies into biological control of mosquitoes and the diseases they spread, for example by *Wolbachia*-based mosquito control techniques. *Wolbachia* infection has been associated with pathogen blocking in several species of mosquitoes (55–57) and with reduced *Ae. aegypti* lifespan and egg viability (58, 59). One hypothesized mechanism is that *Wolbachia* modifies host lipid content, leading to pathogen blocking (60–62). Therefore, the data from this study may also provide a foundation for studies investigating the mechanisms of how *Wolbachia* interacts with host lipid metabolic pathways, which can further broaden our understanding of the important role that lipids play in mosquito physiology and how their metabolism can be used for new pest control strategies.

## Data Availability Statement

The original contributions presented in the study are included in the article/Supplementary Material, further inquiries can be directed to the corresponding author/s.

## Author Contributions

MP designed experiments, performed Nile Red staining and confocal microscopy, performed glycerolipid assays, performed lipid extractions and lipidomics assays, analyzed lipidomics data, generated figures, and wrote the manuscript. SM performed Nile Red staining, analyzed Nile Red images, performed glycerolipid assays, and performed lipid extractions. SR reared mosquitoes and analyzed Nile Red images. YL performed statistical analysis of glycerolipid and Nile Red assay data, analyzed lipidomics data, and generated figures. YK dissected and imaged midguts and ovaries for Figure 1, performed lipid extractions and assisted with lipidomics assays. BD assisted with lipidomics assays. FOH designed experiments, provided equipment and reagents for lipidomics assays, and wrote the manuscript. GA designed experiments, analyzed lipidomics data, and generated figures. IH designed experiments and wrote the manuscript. All authors contributed to the article and approved the submitted version.

## Conflict of Interest

The authors declare that the research was conducted in the absence of any commercial or financial relationships that could be construed as a potential conflict of interest.
