# Supplementary material for: Fat and Happy: Profiling Mosquito Fat Body Lipid Storage and Composition Post-blood Meal
Source: Front Insect Sci. 2021 Jun 16;1:693168. doi: 10.3389/finsc.2021.693168 (PMC10926494; doi:10.3389/finsc.2021.693168)
Supplement: Supplementary file 2 [file Data_Sheet_1.PDF]

Supplemental File 2. WGCNA analysis of lipids to identify clusters of lipids changing similarly during vitellogenesis. A. Dendrogram of lipids with clusters formed by similar changes in abundance during vitellogenesis. The colored bar below represents modules, with colors denoting lipids that change in similar patterns during the vitellogenic cycle. B. Isolation of the three largest modules for part A showing the patterns of change during vitellogenesis. The colors of the module titles represent the modules from part A, while the colors of the graph correspond to the different lipid classes in the key. Many phospholipids are clustered together in the “blue” module. The “brown” module consists of neutral lipids, DAG and TAG. Finally, the “turquoise” module contains a small amount of TAGs and larger amounts of phospholipids and lysophospholipids.

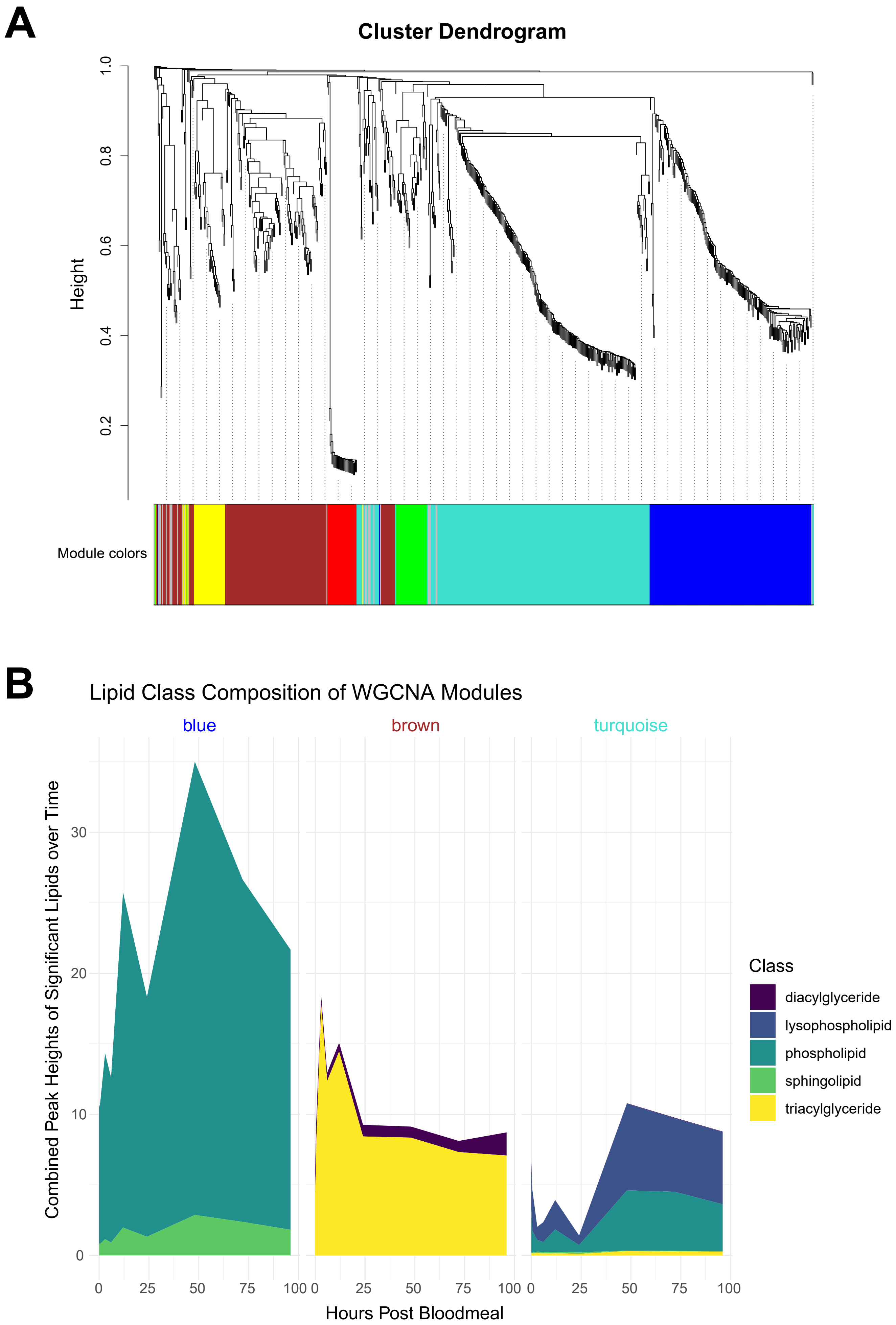
A
Cluster Dendrogram
Height
1.0
0.8
0.6
0.4
0.2
Module colors
B
Lipid Class Composition of WGCNA Modules
blue
brown
turquoise
Combined Peak Heights of Significant Lipids over Time
30
20
10
0
0 25 50 75 100
Hours Post Bloodmeal
Class
diacylglyceride
lysophospholipid
phospholipid
sphingolipid
triacylglyceride
