# Supplementary material for: Fat and Happy: Profiling Mosquito Fat Body Lipid Storage and Composition Post-blood Meal
Source: Front Insect Sci. 2021 Jun 16;1:693168. doi: 10.3389/finsc.2021.693168 (PMC10926494; doi:10.3389/finsc.2021.693168)
Supplement: Supplementary file 3 [file Data_Sheet_2.PDF]

Supplemental File 3. Bar graphs illustrating changes in non-glycerolipid lipids identified by LC/MS over the vitellogenic cycle. Lipid subclasses in these graphs were identified in MS-DIAL and manually re-annotated using LipidMaps. Abbreviations: Cer - ceramide; SM - sphingomyelin; SPB - sphinganine; CE- cholesteryl ester; CL - cardiolipin; WE - wax ester. Data are represented as means ± SEM. Statistically significant changes were determined using Kruskal-Wallis tests, and letters represent statistically significant differences between time points ($p<0.05$). Note, the scale on the y-axis differs on each panel and represents the sum of normalized peak heights for all lipids within the given lipid class. 0 hr PBM time point represents unfed mosquitoes. Lipid drawings are example structures.

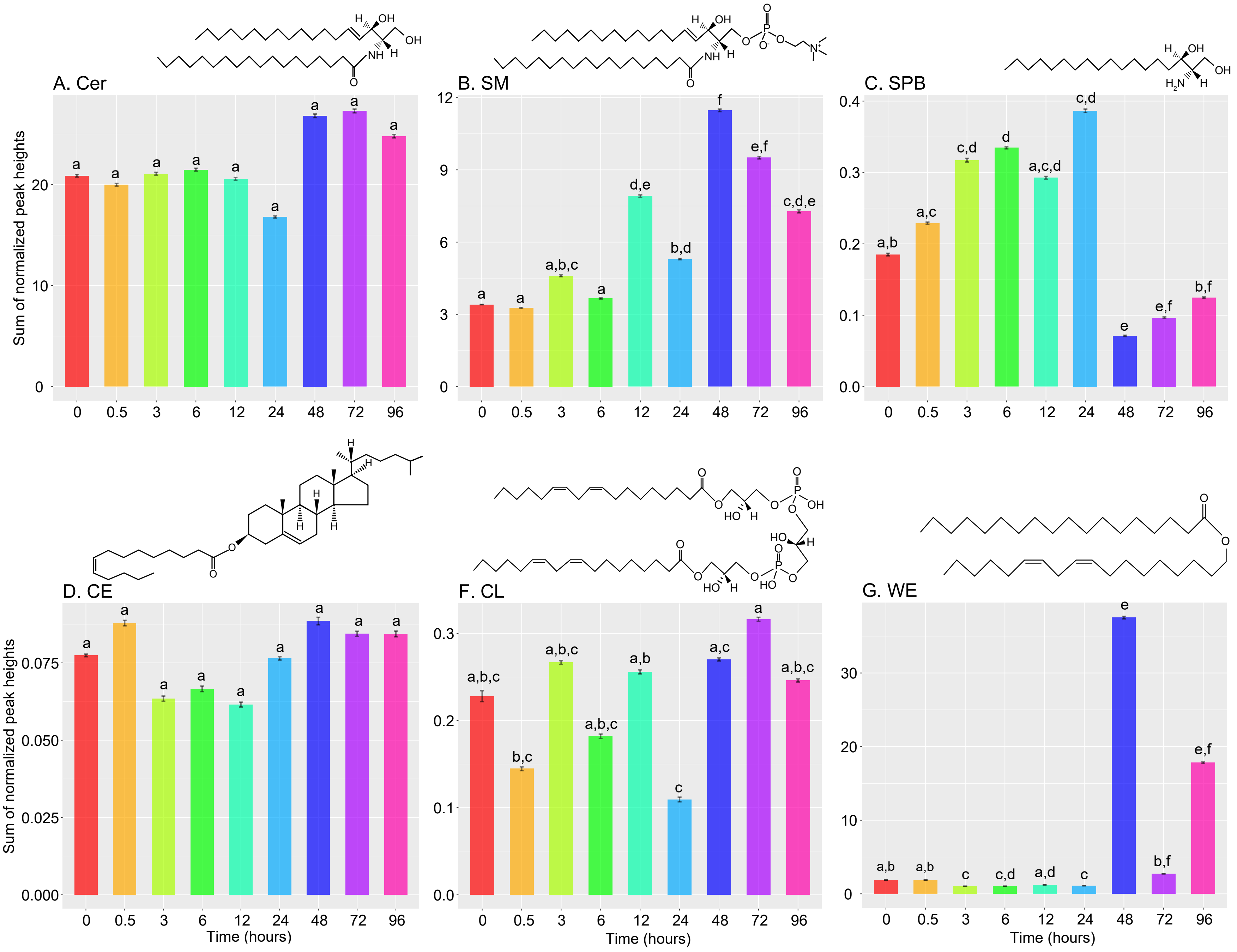

A. Cer
B. SM
C. SPB
D. CE
F. CL
G. WE
Sum of normalized peak heights
Time (hours)
0
0.5
3
6
12
24
48
72
96
OH
NH
H
O
P
O-
N+
H2N
HO
a
a,b
a,c
b,c
c
c,d
d
a,c,d
e
e,f
b,f
a,b,c
d,e
b,d
f
c,d,e
a,b
a,c
a,d
