# Supplementary material for: Fat and Happy: Profiling Mosquito Fat Body Lipid Storage and Composition Post-blood Meal
Source: Front Insect Sci. 2021 Jun 16;1:693168. doi: 10.3389/finsc.2021.693168 (PMC10926494; doi:10.3389/finsc.2021.693168)
Supplement: Supplementary file 4 [file Data_Sheet_3.PDF]

Supplemental File 4. Boxplots of individual lipids which changed significantly over the vitellogenic cycle. Manually re-annotated lipids which changed significantly were determined using Spearman correlation coefficients. Categories represent the specific lipid identified, and id values represent the peak IDs from the lipidome dataset in supplemental file 1. Black lines represent median normalized peak heights at each time point. Boxes illustrate 2nd-3rd interquartile range, and whiskers represent 1st and 4th quartiles. Circles represent outliers. Note, the scale on the y-axis differs on each graph. 0 hr PBM time point represents unfed mosquitoes.

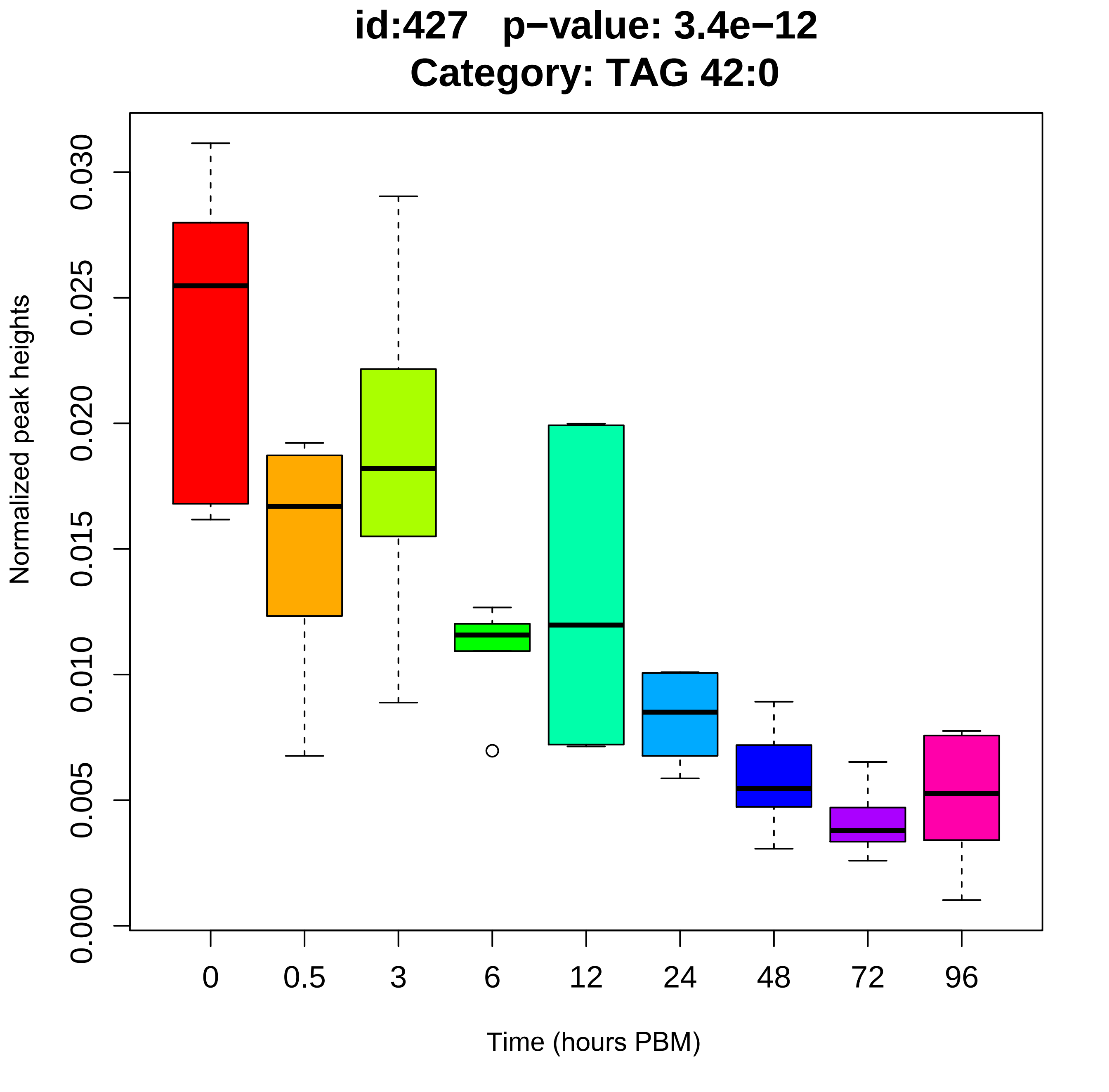

id:427 p−value: 3.4e−12
Category: TAG 42:0
Normalized peak heights
0.000
0.005
0.010
0.015
0.020
0.025
0.030
0
0.5
3
6
12
24
48
72
96
Time (hours PBM)

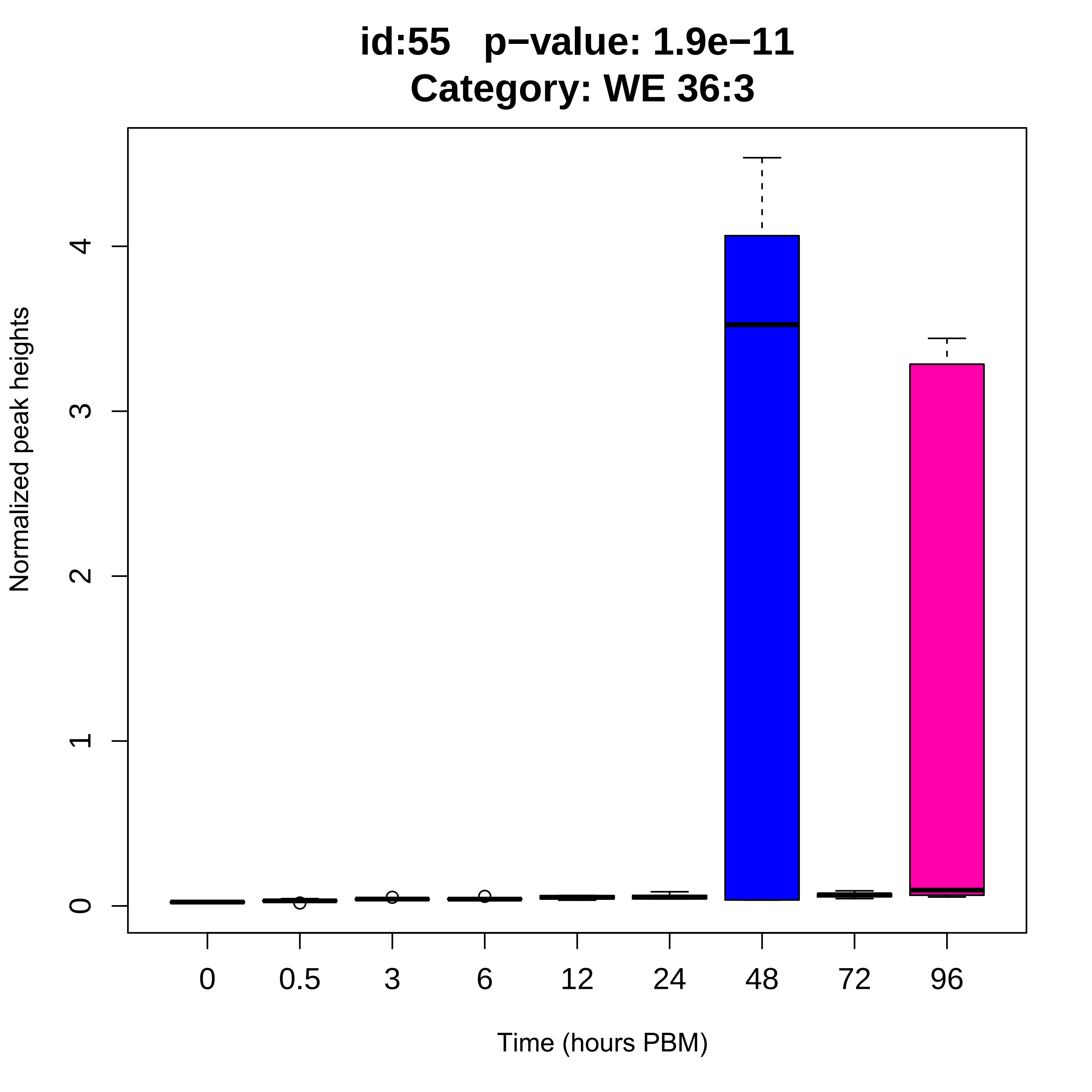

id:55 p−value: 1.9e−11
Category: WE 36:3
Normalized peak heights
0
1
2
3
4
0
0.5
3
6
12
24
48
72
96
Time (hours PBM)

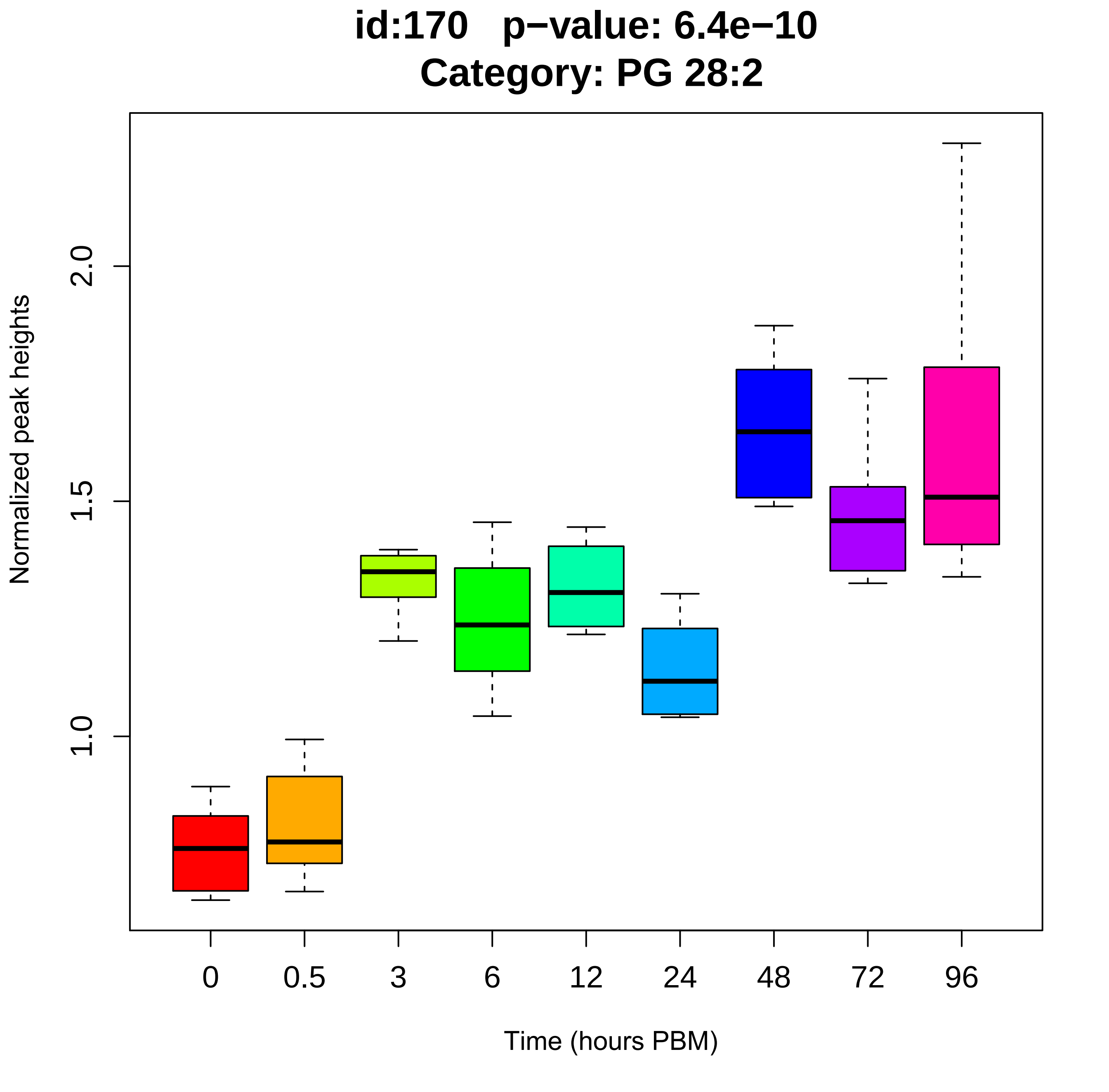
id:170 p−value: 6.4e−10
Category: PG 28:2
Normalized peak heights
2.0
1.5
1.0
0
0.5
3
6
12
24
48
72
96
Time (hours PBM)

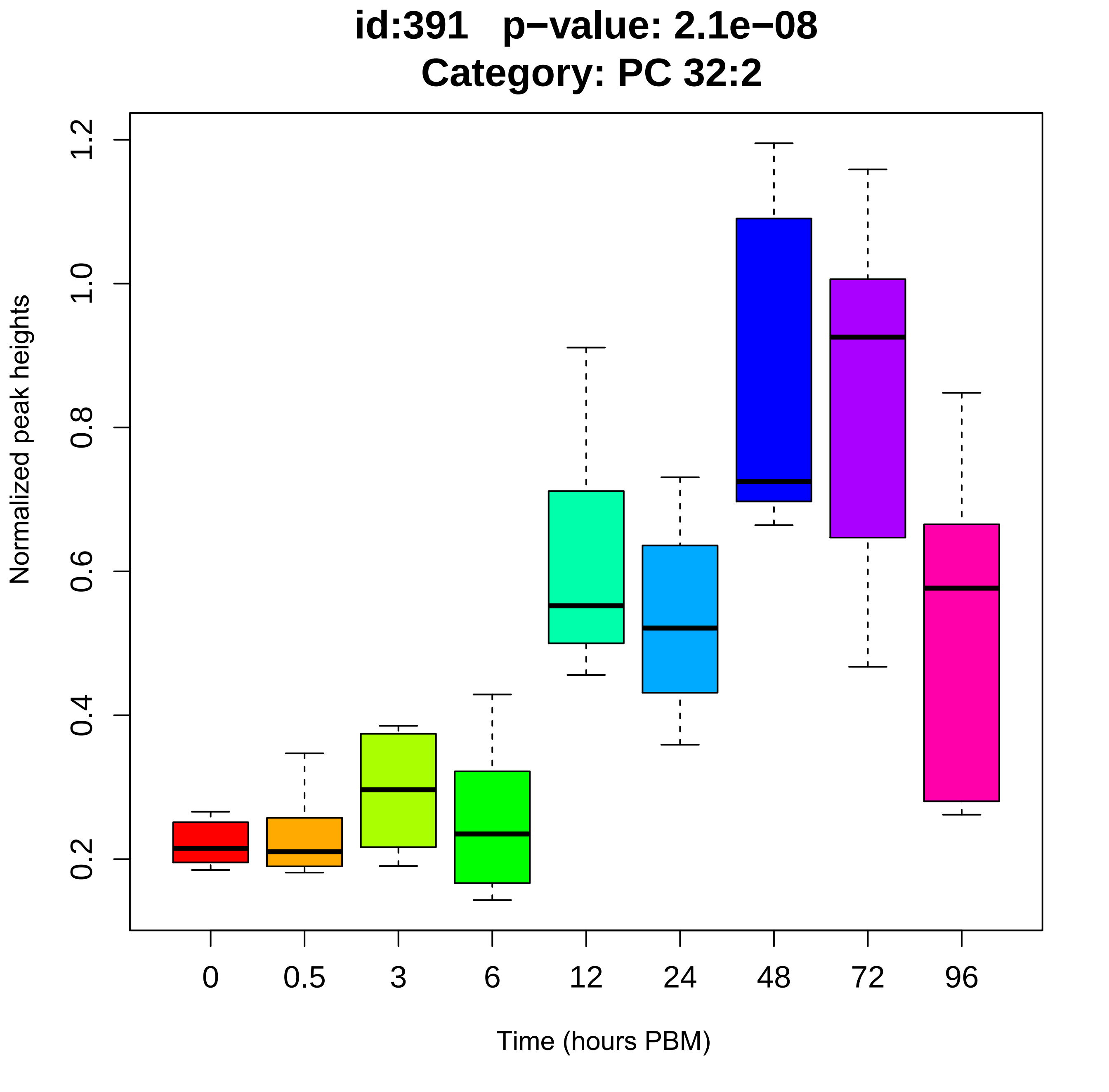

id:391 p−value: 2.1e−08
Category: PC 32:2
Normalized peak heights
0.2
0.4
0.6
0.8
1.0
1.2
0
0.5
3
6
12
24
48
72
96
Time (hours PBM)

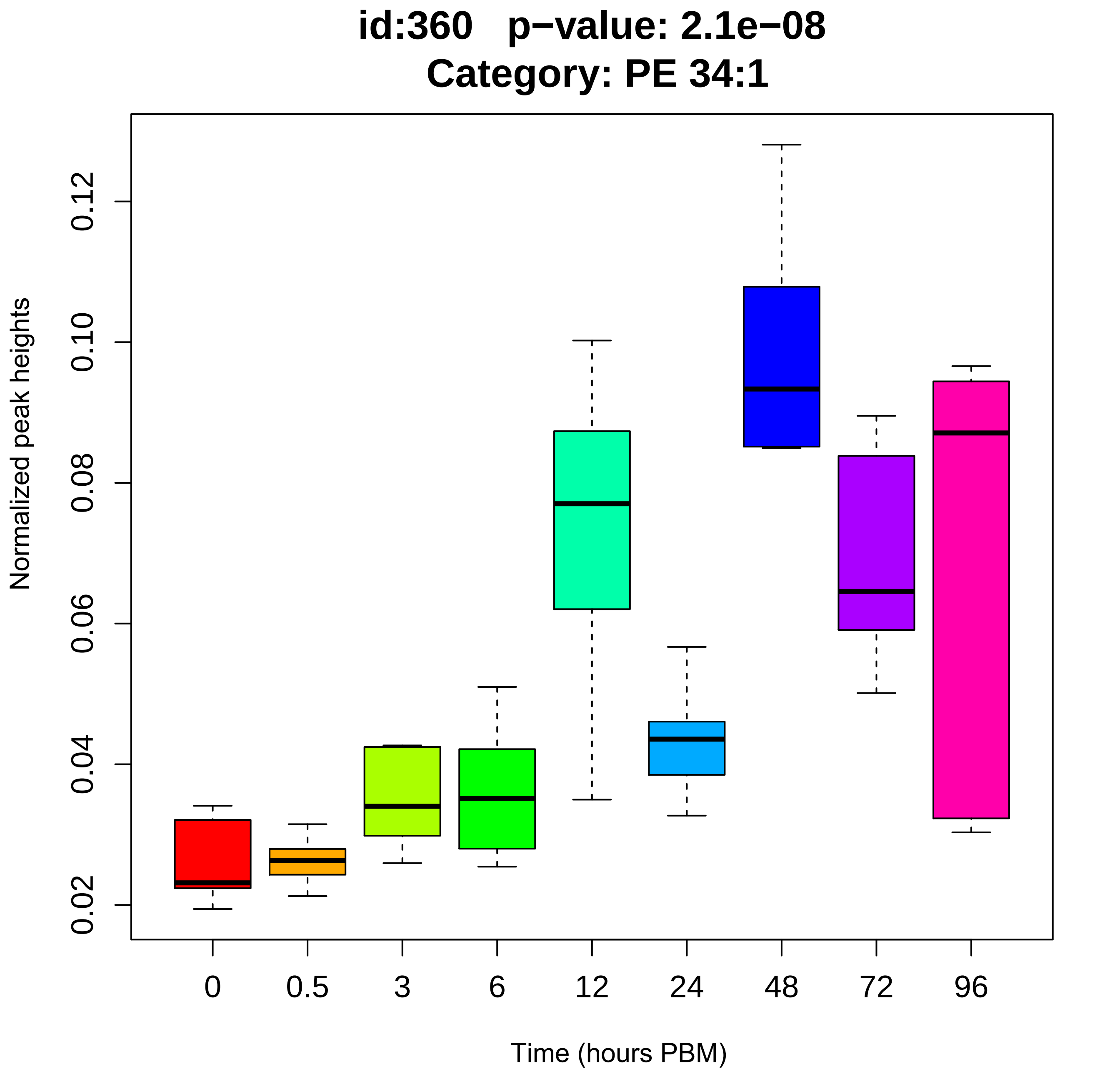
id:360 p−value: 2.1e−08
Category: PE 34:1
Normalized peak heights
0.02
0.04
0.06
0.08
0.10
0.12
0
0.5
3
6
12
24
48
72
96
Time (hours PBM)

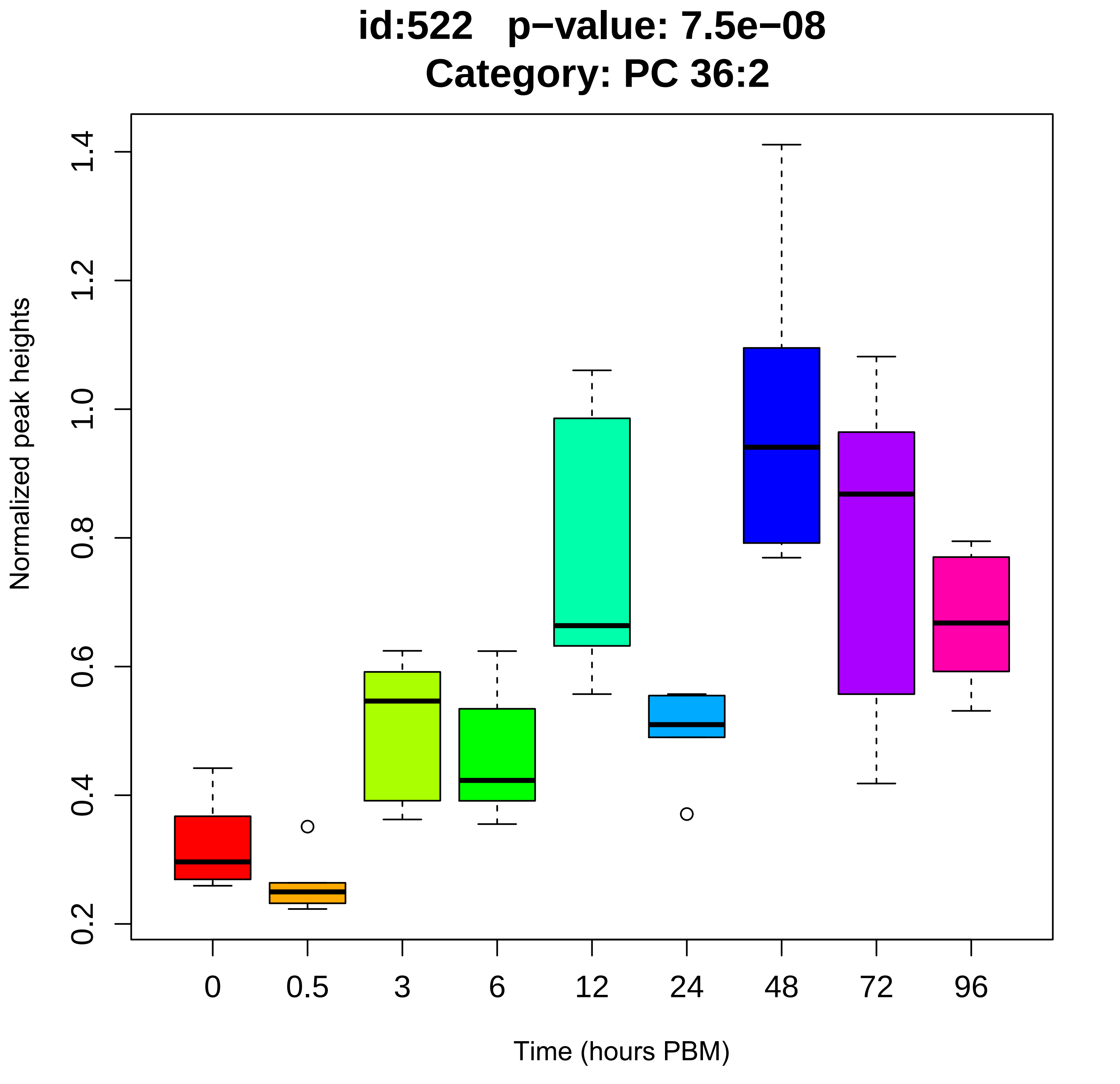
id:522 p−value: 7.5e−08
Category: PC 36:2
Normalized peak heights
0.2
0.4
0.6
0.8
1.0
1.2
1.4
0
0.5
3
6
12
24
48
72
96
Time (hours PBM)

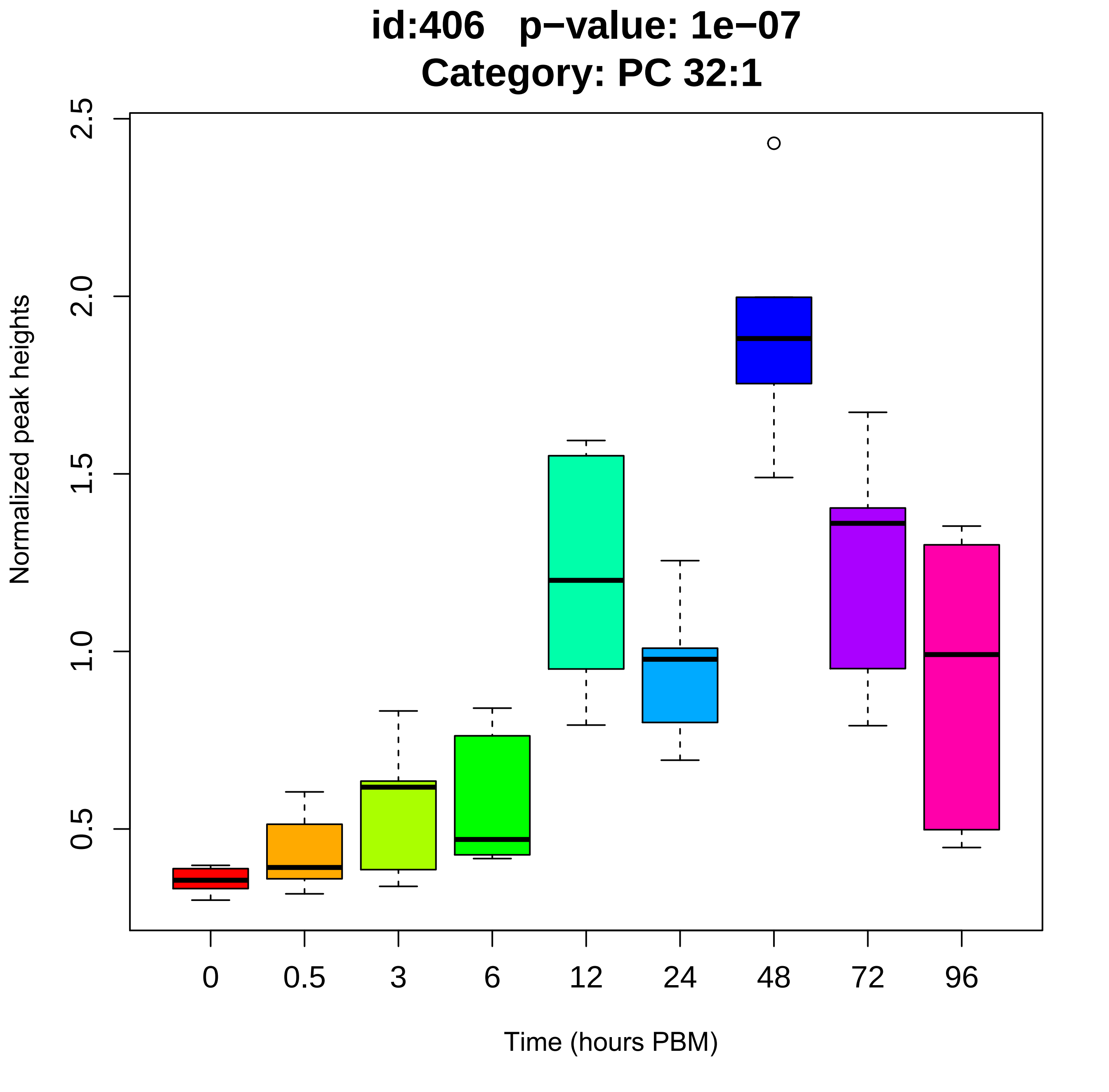

id:406 p−value: 1e−07
Category: PC 32:1
Normalized peak heights
0.5
1.0
1.5
2.0
2.5
0
0.5
3
6
12
24
48
72
96
Time (hours PBM)

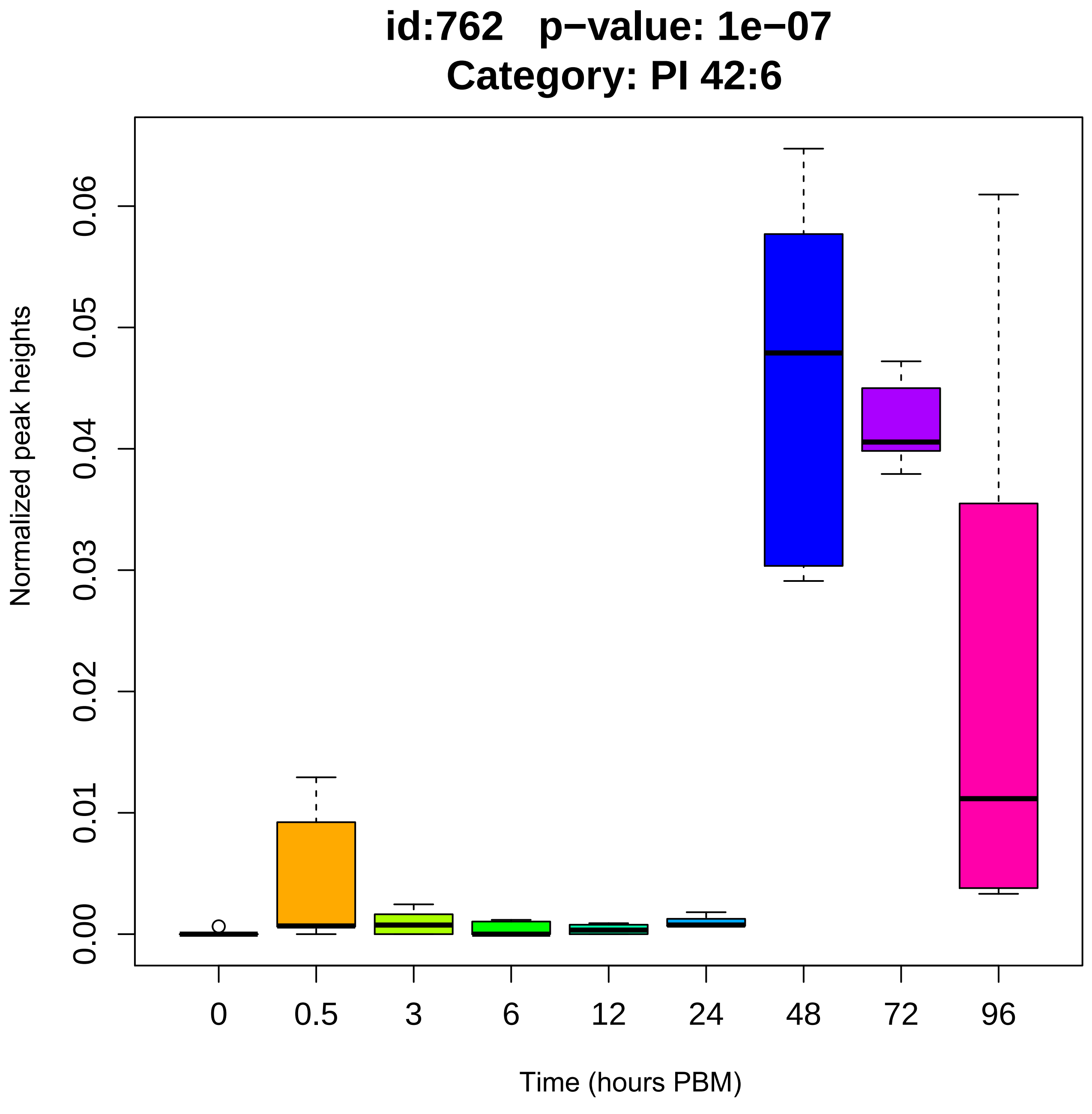

id:762 p−value: 1e−07
Category: PI 42:6
Normalized peak heights
0.00
0.01
0.02
0.03
0.04
0.05
0.06
0
0.5
3
6
12
24
48
72
96
Time (hours PBM)

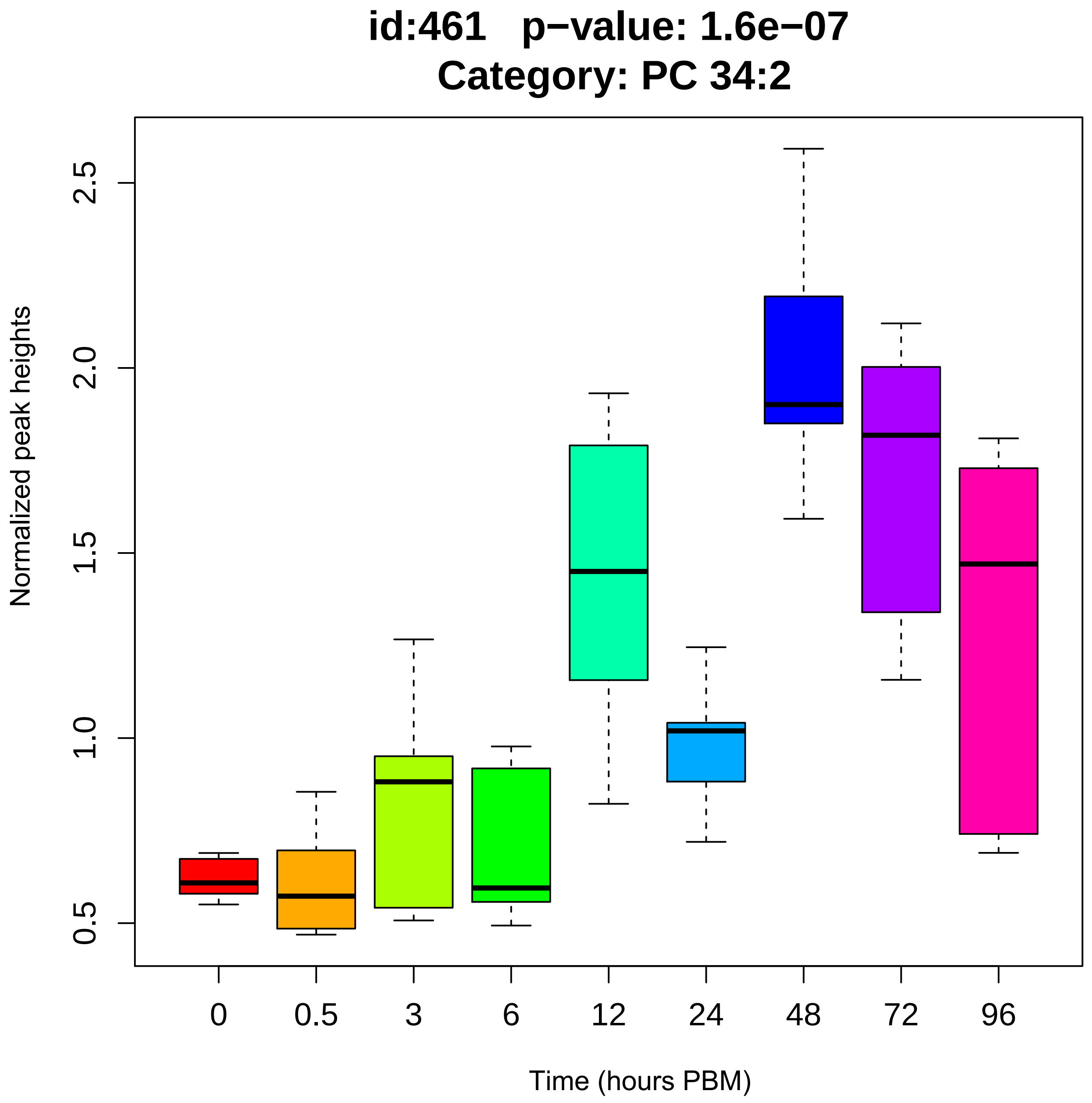

id:461 p−value: 1.6e−07
Category: PC 34:2
Normalized peak heights
0.5
1.0
1.5
2.0
2.5
0
0.5
3
6
12
24
48
72
96
Time (hours PBM)

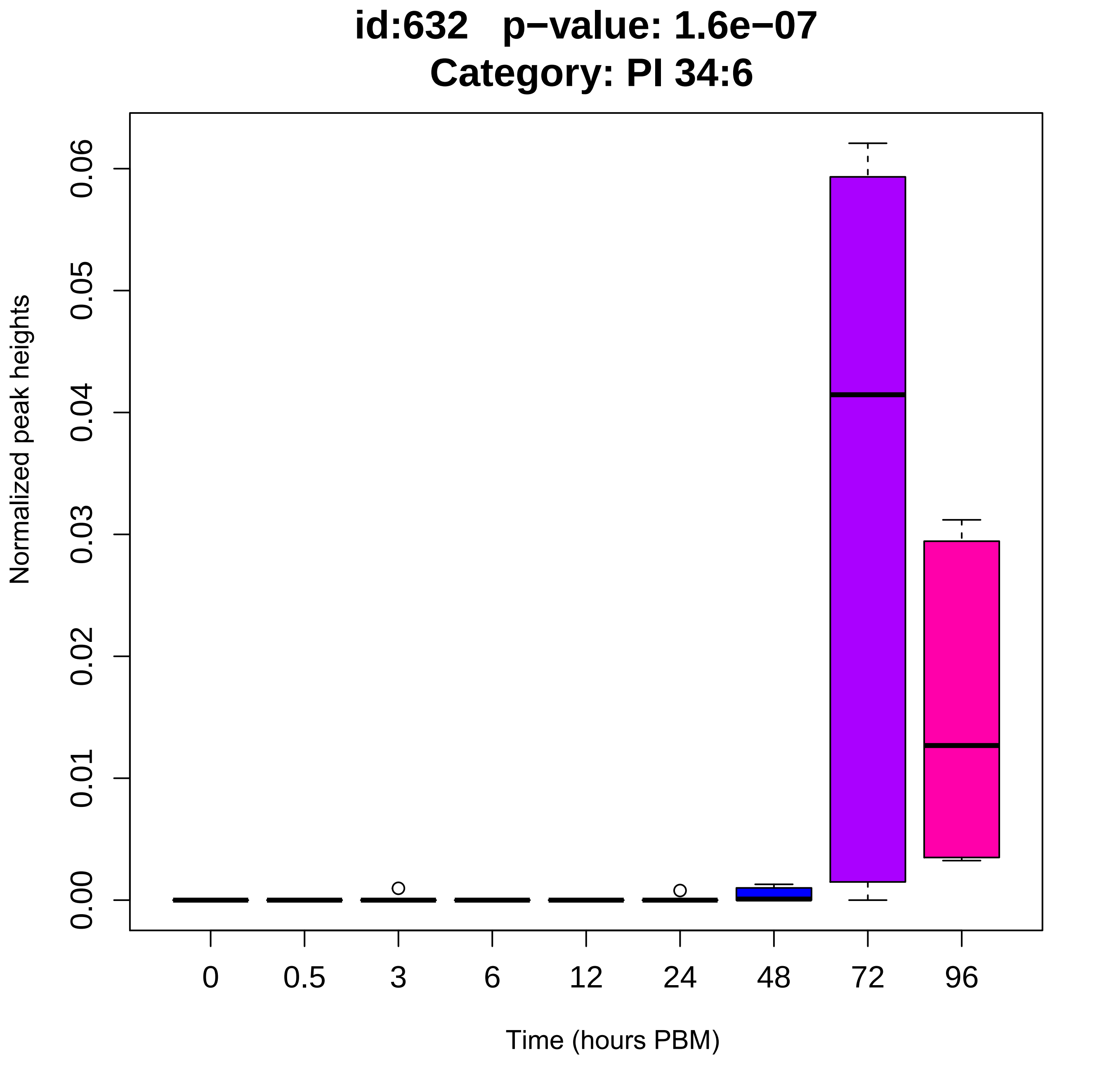
id:632   p−value: 1.6e−07
Category: PI 34:6
Normalized peak heights
0.00
0.01
0.02
0.03
0.04
0.05
0.06
0
0.5
3
6
12
24
48
72
96
Time (hours PBM)

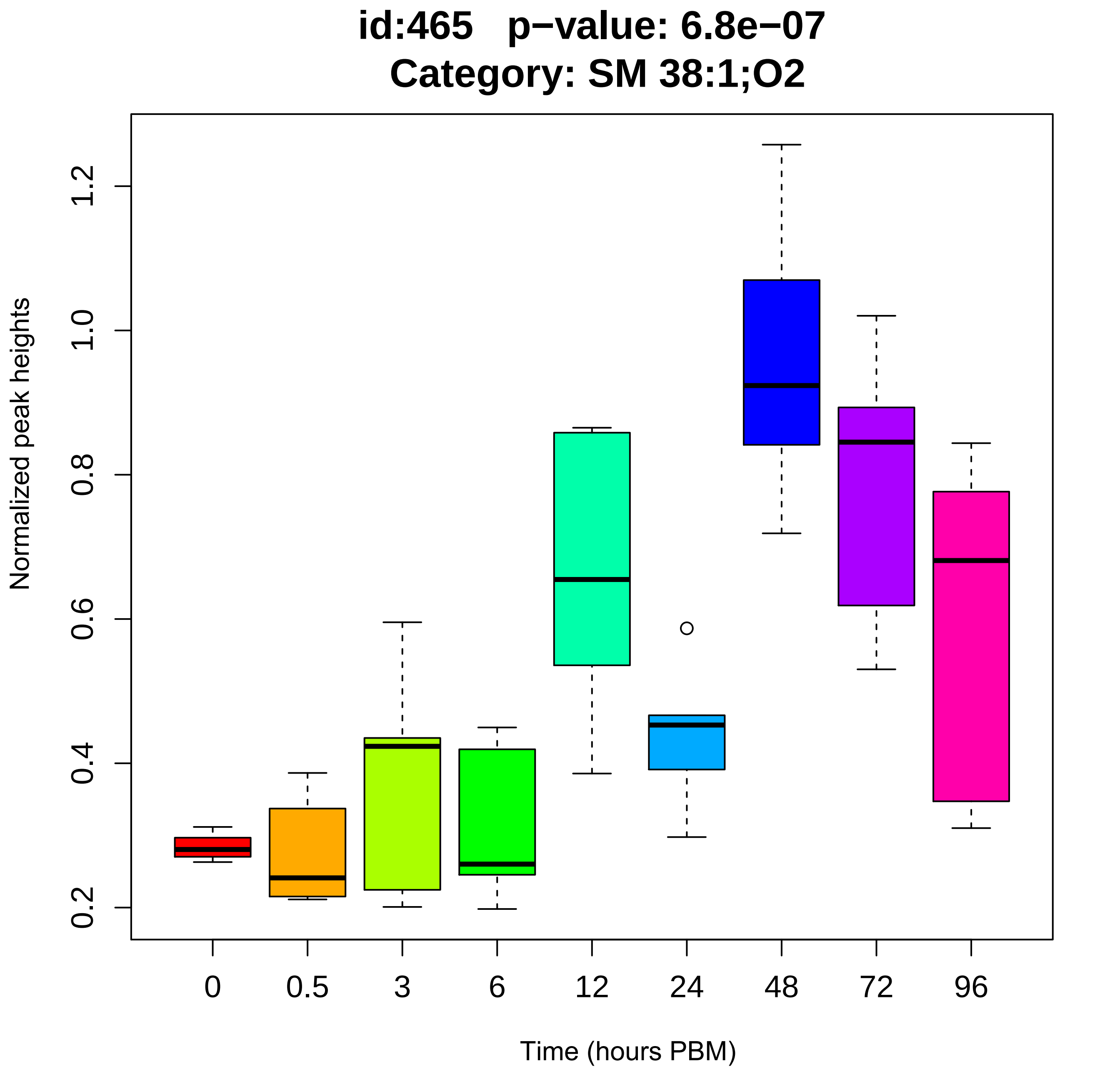

id:465 p−value: 6.8e−07
Category: SM 38:1;O2
Normalized peak heights
0.2
0.4
0.6
0.8
1.0
1.2
0
0.5
3
6
12
24
48
72
96
Time (hours PBM)

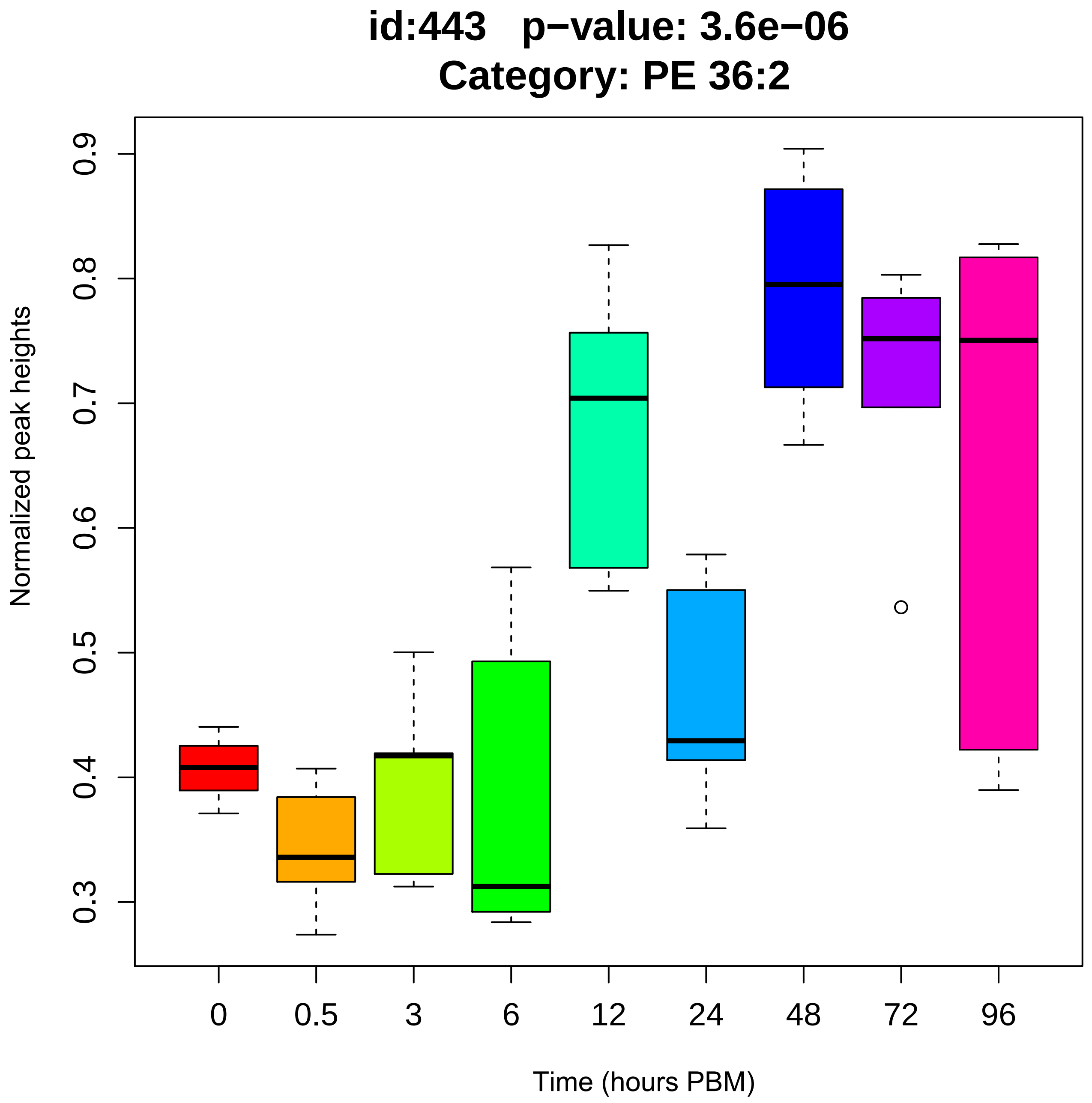
id:443 p−value: 3.6e−06
Category: PE 36:2
Normalized peak heights
0.3
0.4
0.5
0.6
0.7
0.8
0.9
0
0.5
3
6
12
24
48
72
96
Time (hours PBM)

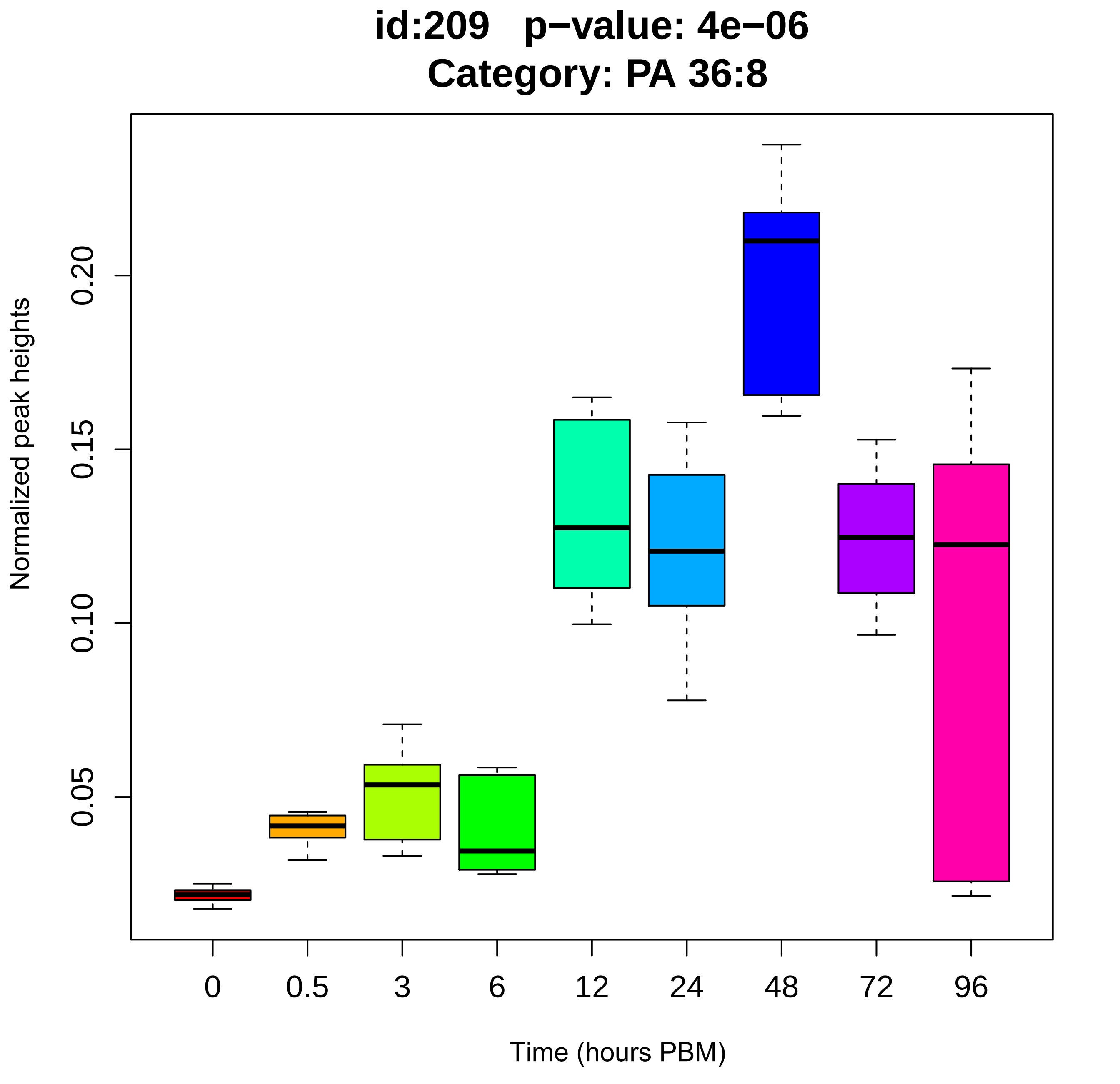
id:209 p−value: 4e−06
Category: PA 36:8
Normalized peak heights
0.05
0.10
0.15
0.20
0
0.5
3
6
12
24
48
72
96
Time (hours PBM)

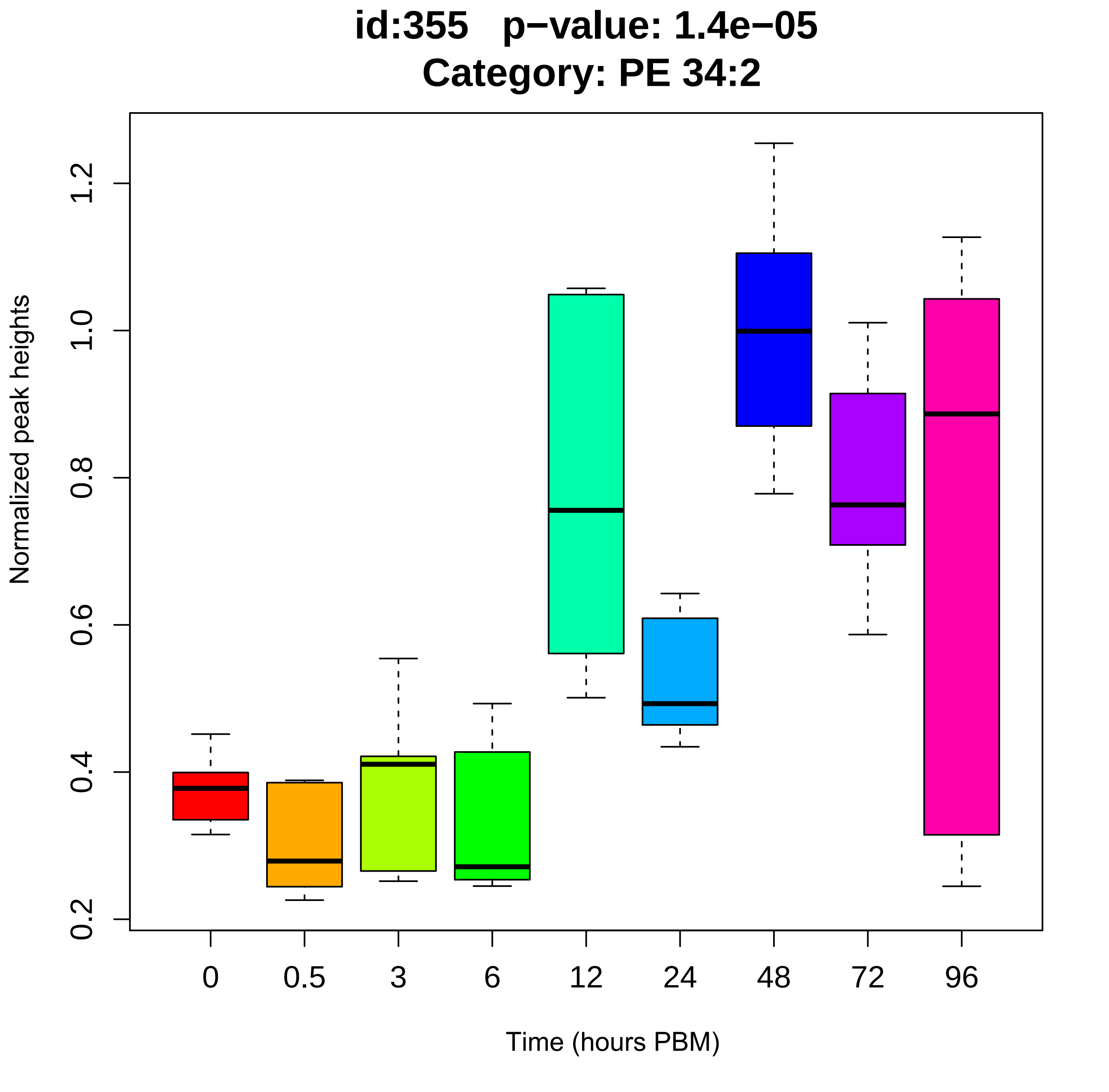
id:355 p−value: 1.4e−05
Category: PE 34:2
Normalized peak heights
0.2
0.4
0.6
0.8
1.0
1.2
0
0.5
3
6
12
24
48
72
96
Time (hours PBM)

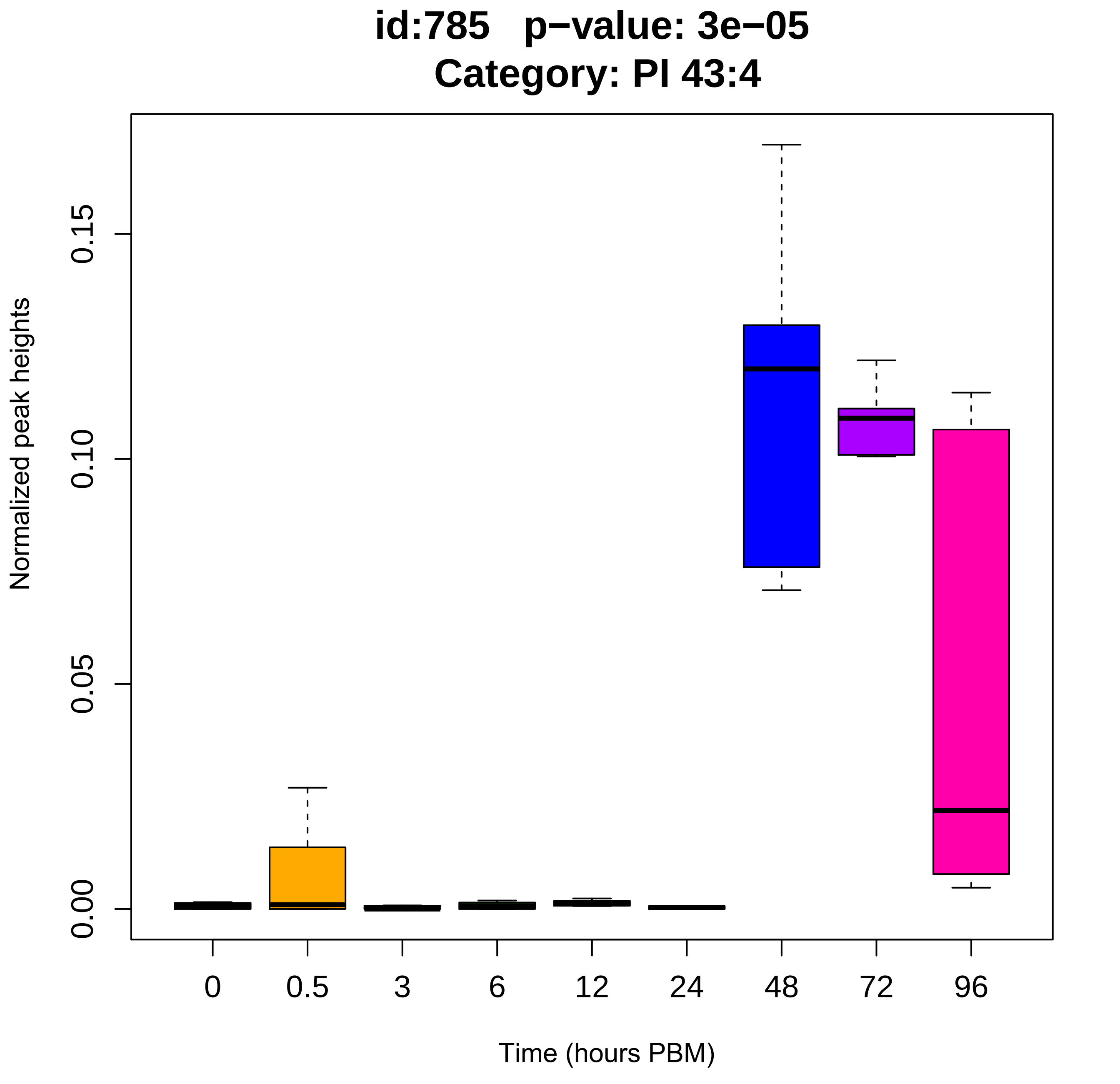
id:785 p−value: 3e−05
Category: PI 43:4
Normalized peak heights
0.00
0.05
0.10
0.15
0
0.5
3
6
12
24
48
72
96
Time (hours PBM)

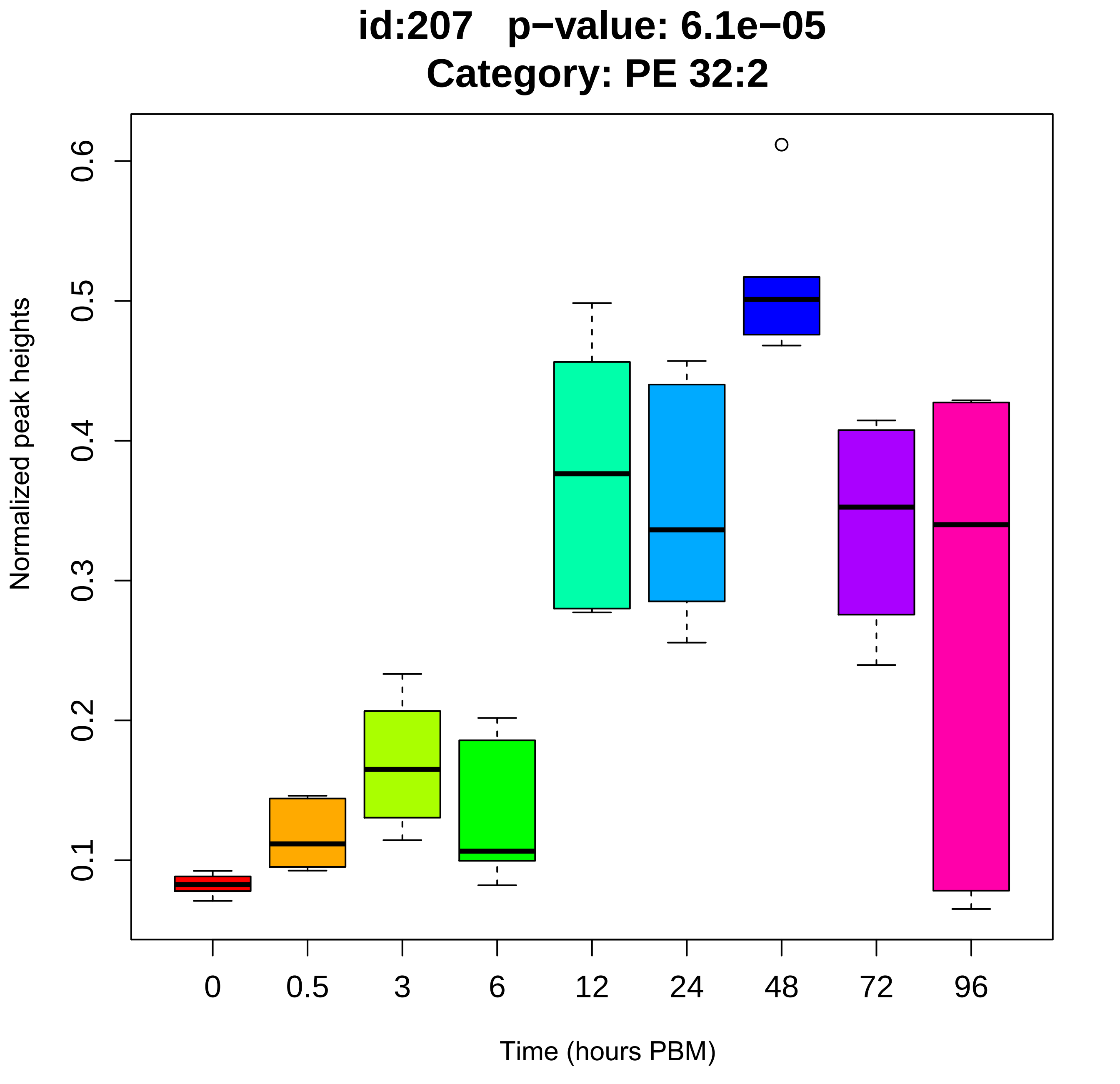
id:207 p−value: 6.1e−05
Category: PE 32:2
Normalized peak heights
0.1
0.2
0.3
0.4
0.5
0.6
0
0.5
3
6
12
24
48
72
96
Time (hours PBM)

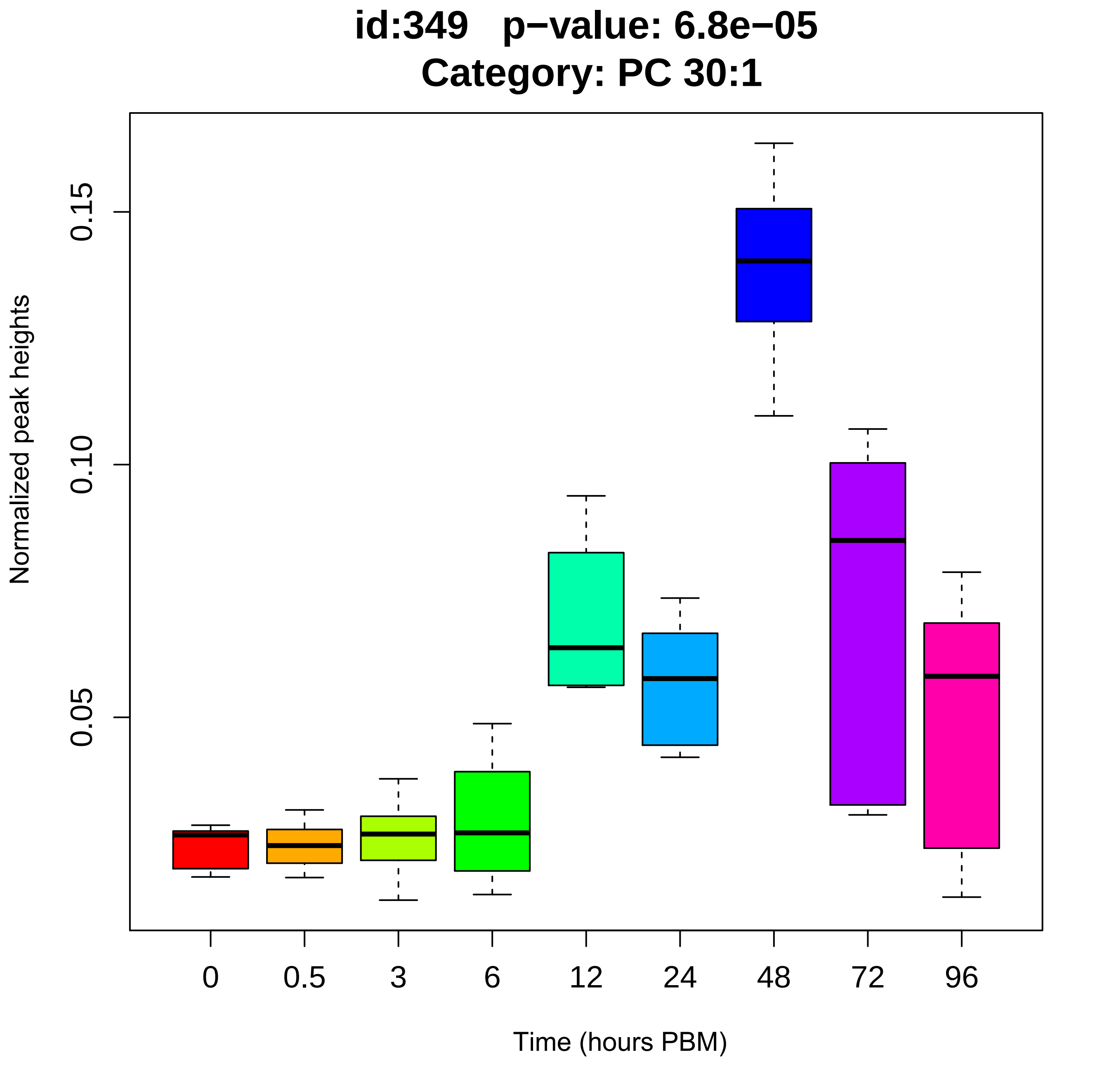

id:349 p−value: 6.8e−05
Category: PC 30:1
Normalized peak heights
0.05
0.10
0.15
0
0.5
3
6
12
24
48
72
96
Time (hours PBM)

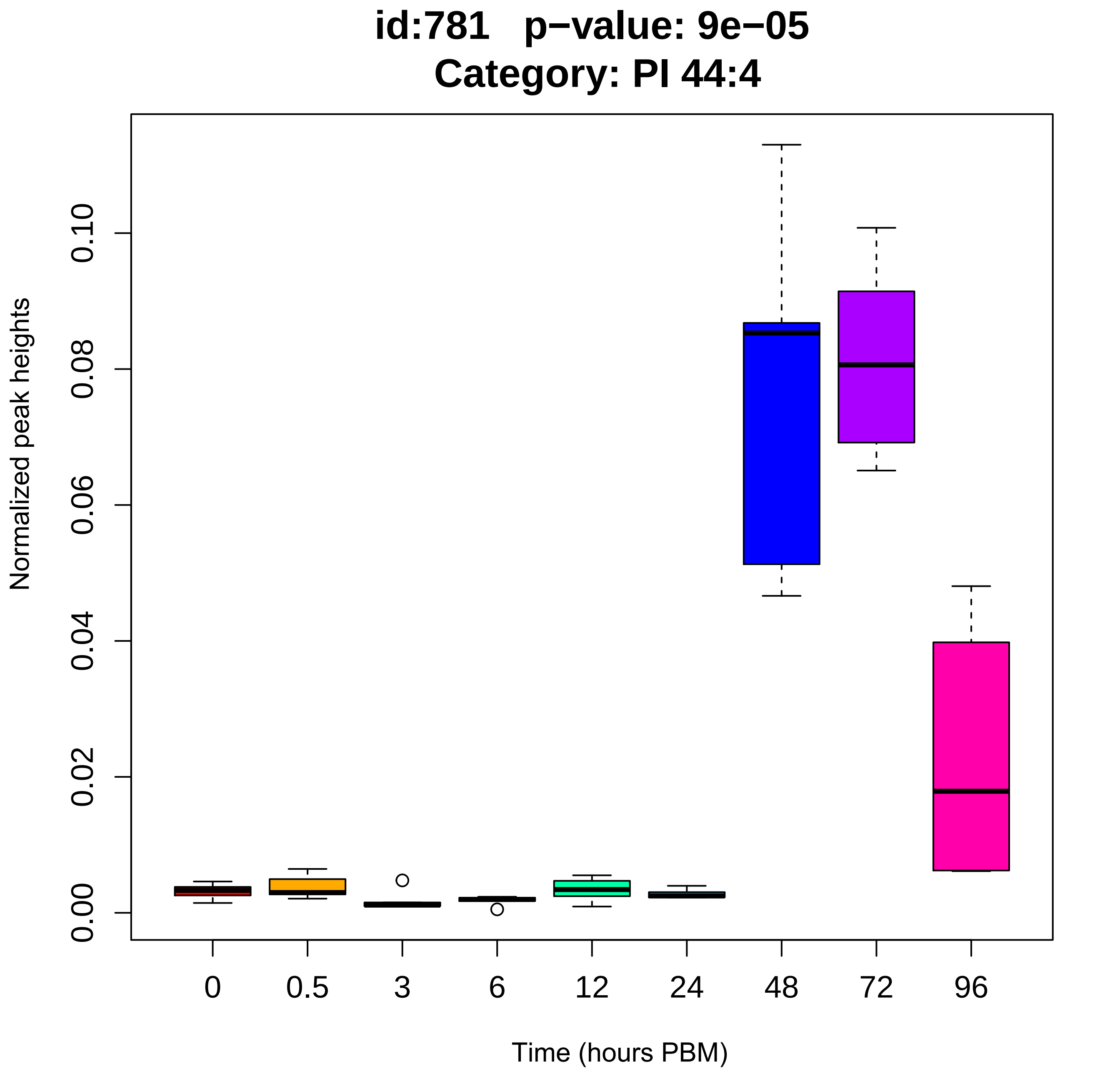

id:781 p−value: 9e−05
Category: PI 44:4
Normalized peak heights
0.00
0.02
0.04
0.06
0.08
0.10
0
0.5
3
6
12
24
48
72
96
Time (hours PBM)

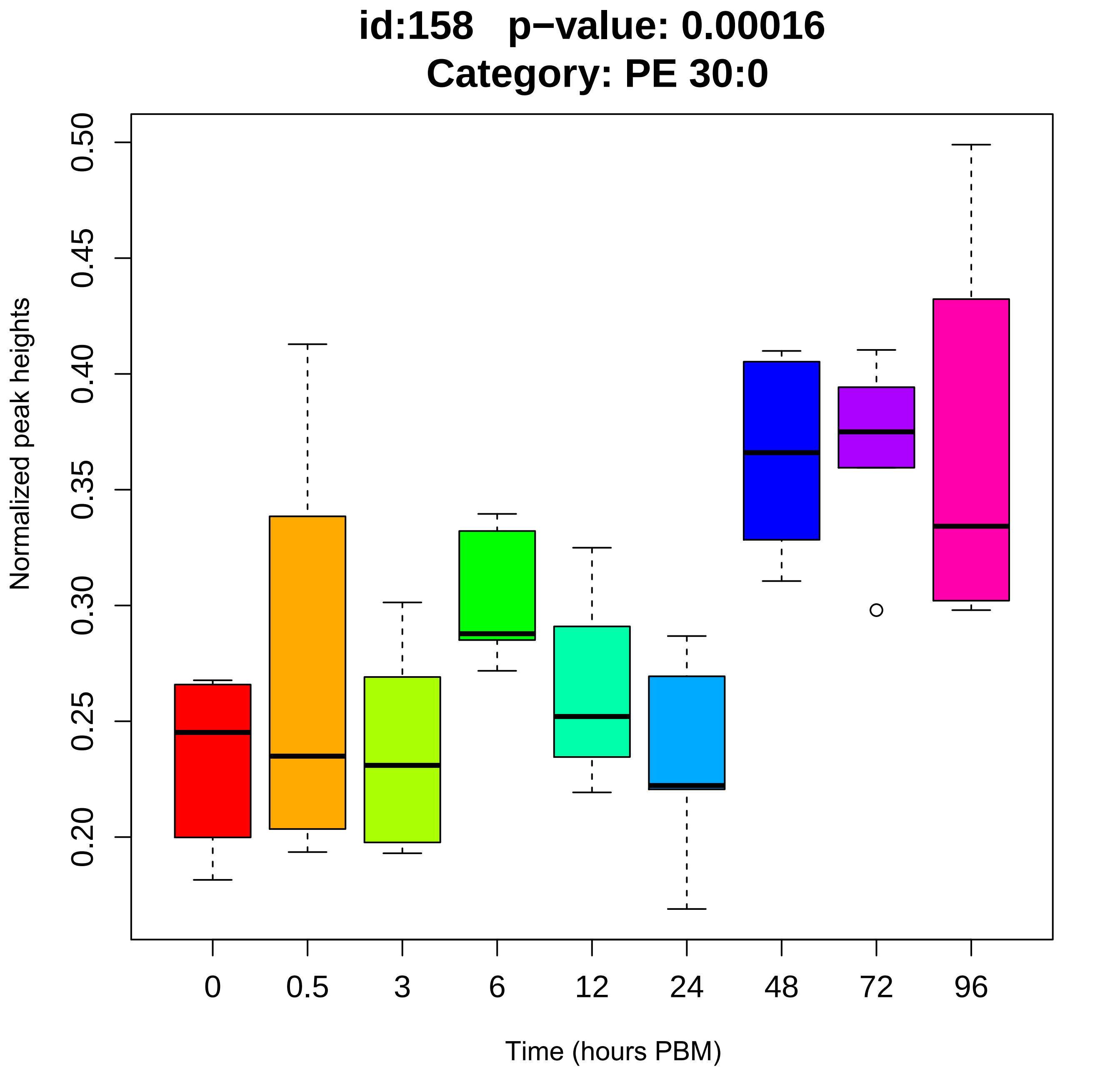
id:158 p−value: 0.00016
Category: PE 30:0
Normalized peak heights
0.20
0.25
0.30
0.35
0.40
0.45
0.50
0
0.5
3
6
12
24
48
72
96
Time (hours PBM)

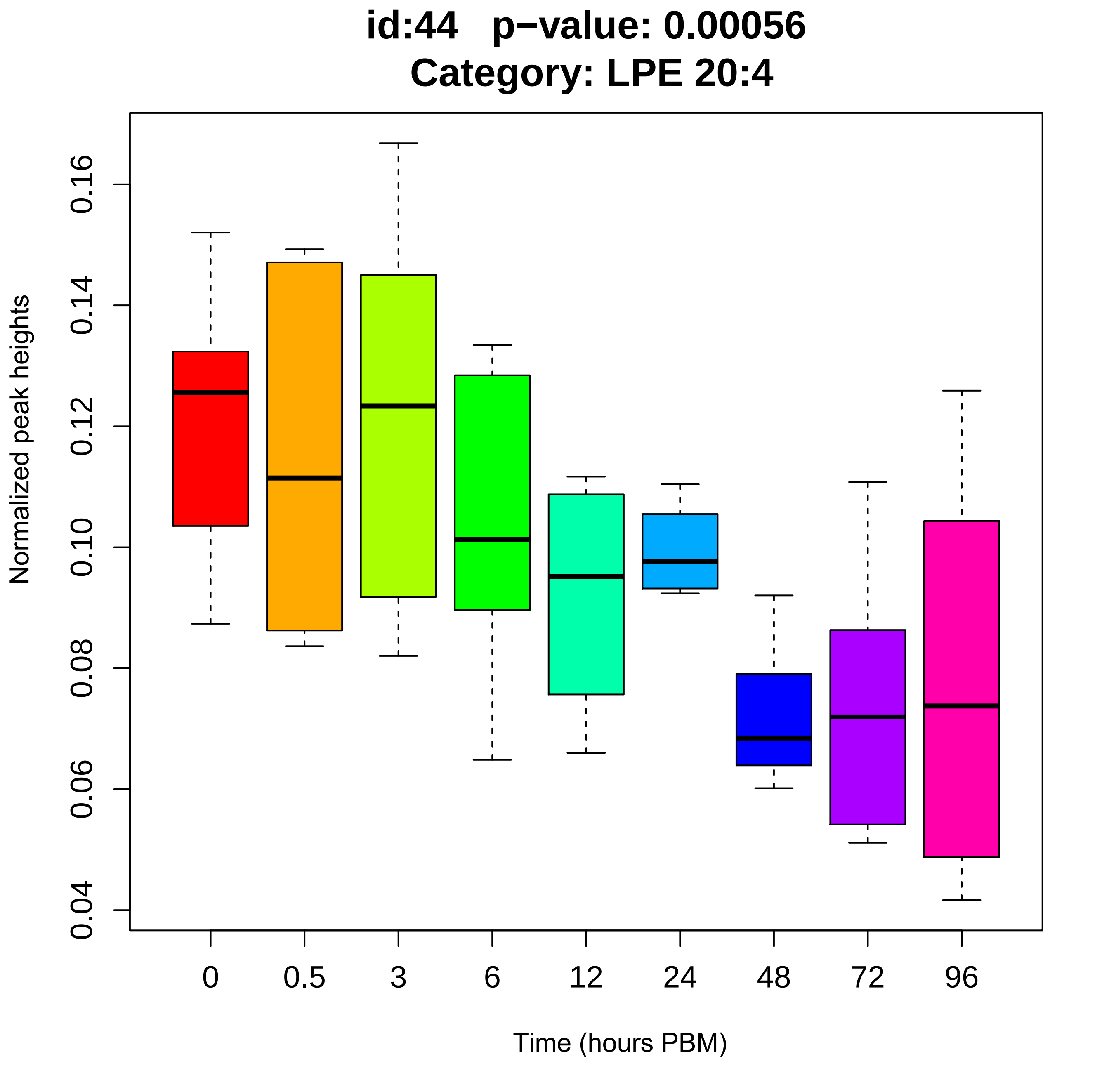
id:44 p−value: 0.00056
Category: LPE 20:4
Normalized peak heights
0.04
0.06
0.08
0.10
0.12
0.14
0.16
0
0.5
3
6
12
24
48
72
96
Time (hours PBM)

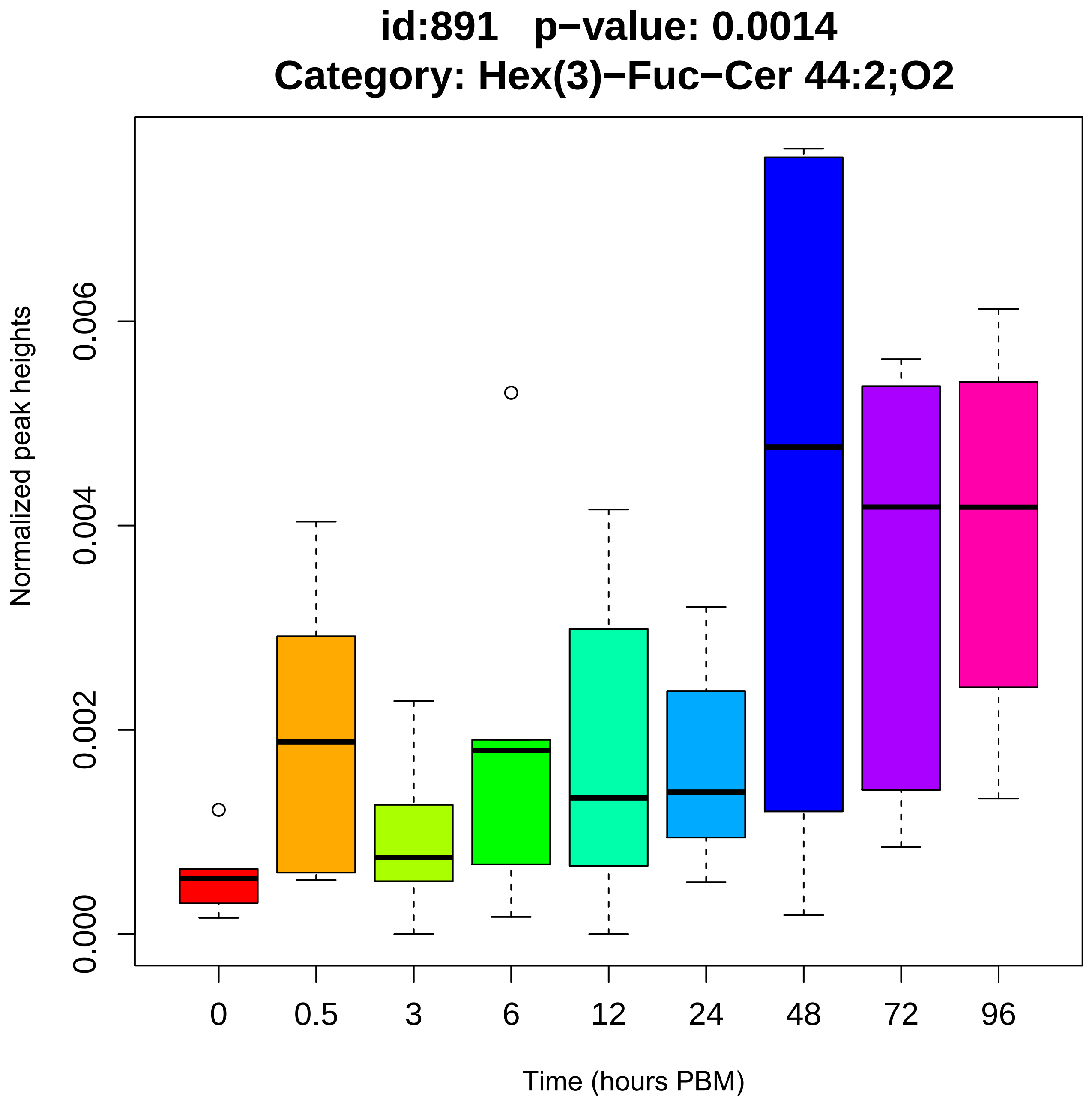

id:891 p−value: 0.0014
Category: Hex(3)−Fuc−Cer 44:2;O2
Normalized peak heights
0.000
0.002
0.004
0.006
0
0.5
3
6
12
24
48
72
96
Time (hours PBM)

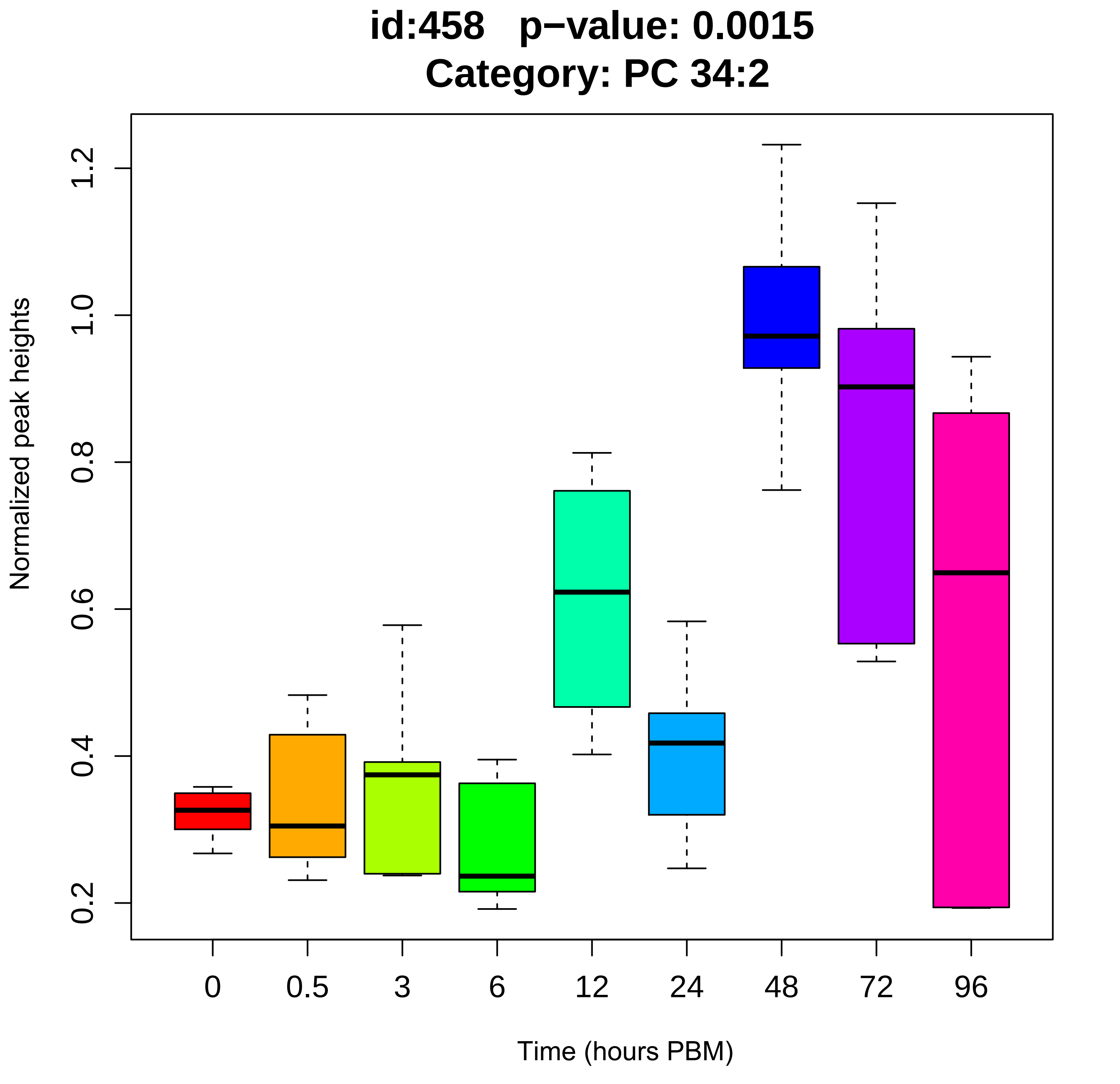

id:458 p−value: 0.0015
Category: PC 34:2
Normalized peak heights
1.2
1.0
0.8
0.6
0.4
0.2
0
0.5
3
6
12
24
48
72
96
Time (hours PBM)

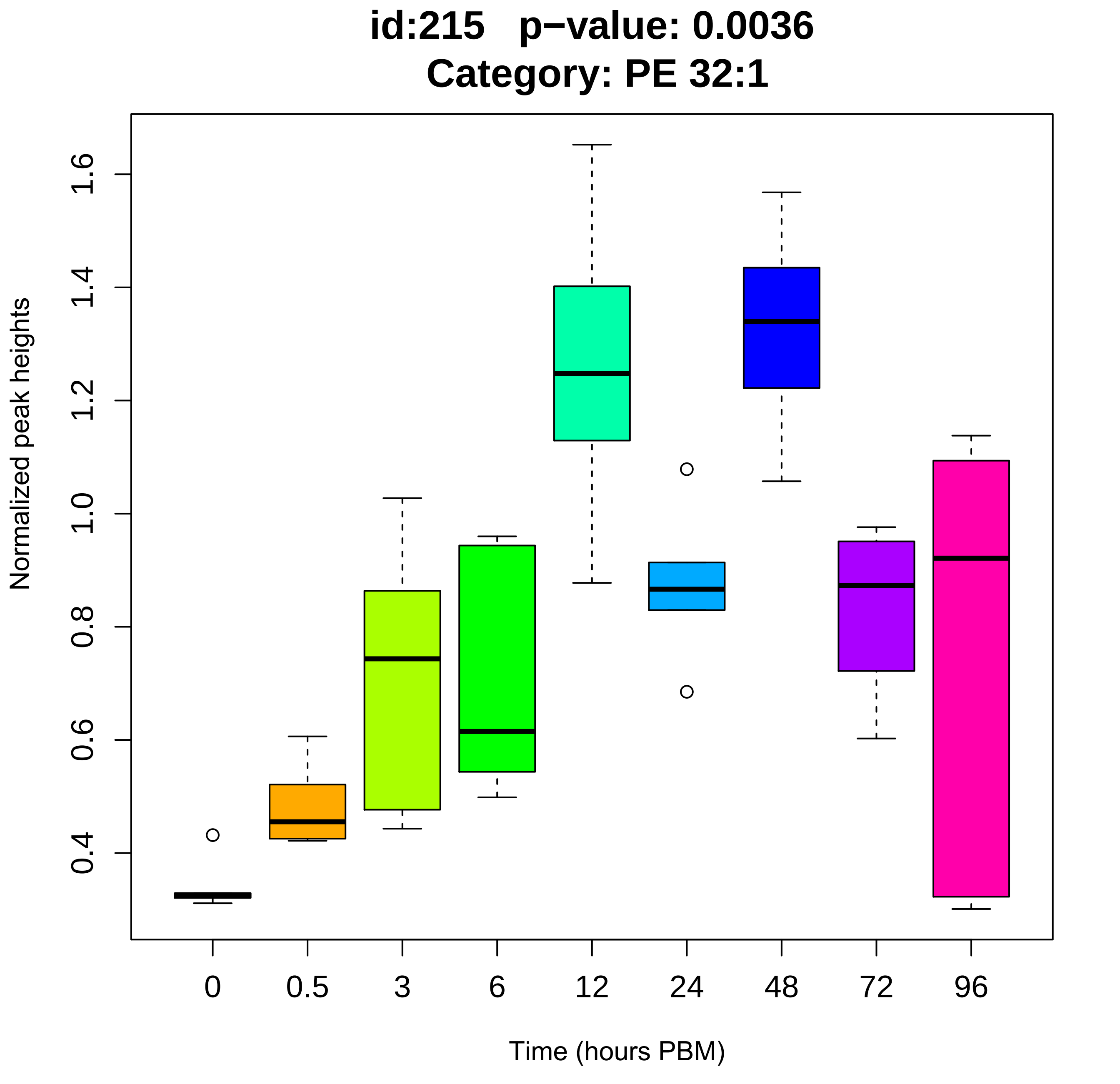
id:215 p−value: 0.0036
Category: PE 32:1
Normalized peak heights
1.6
1.4
1.2
1.0
0.8
0.6
0.4
0
0.5
3
6
12
24
48
72
96
Time (hours PBM)

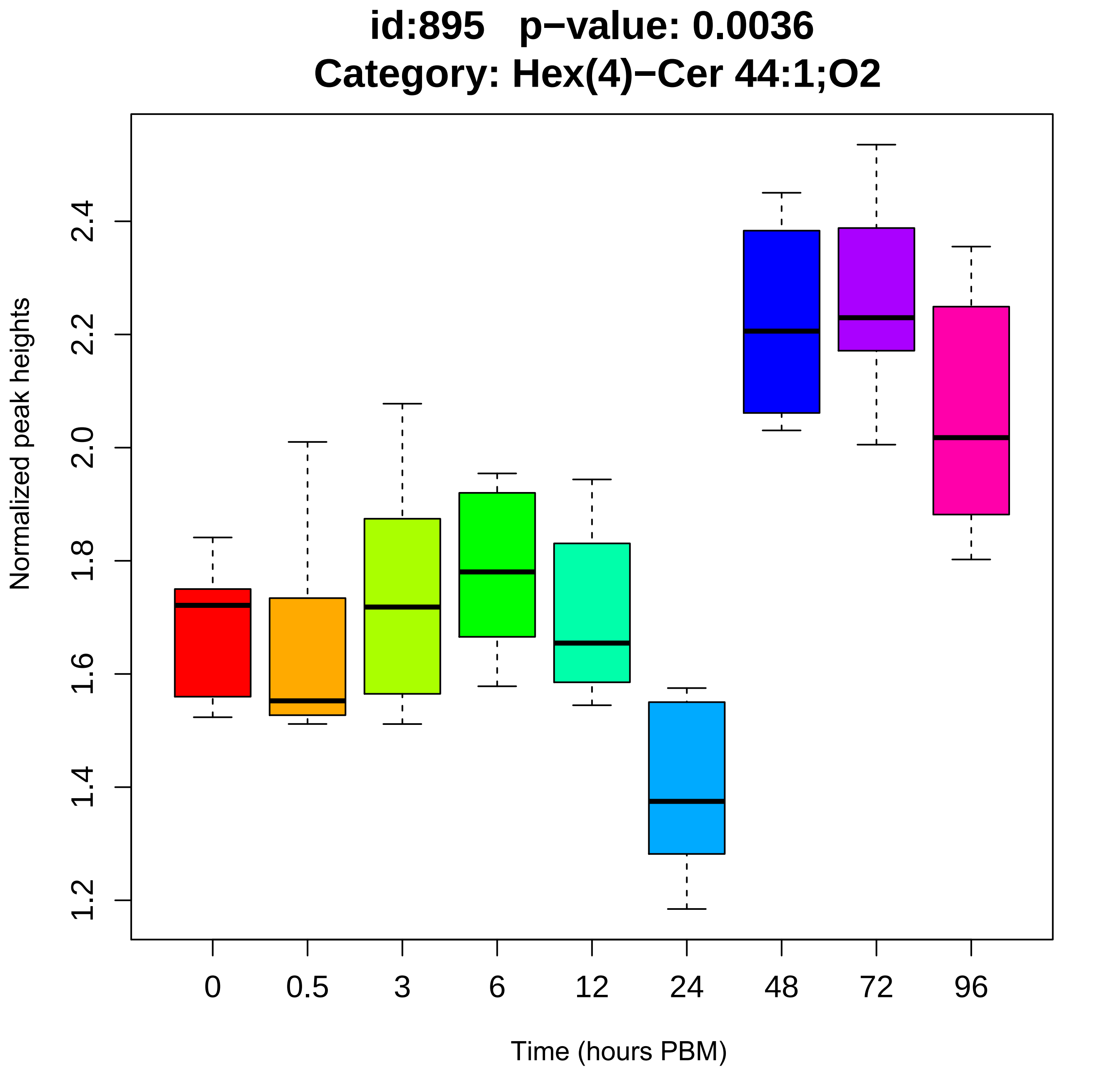

id:895 p−value: 0.0036
Category: Hex(4)−Cer 44:1;O2
Normalized peak heights
1.2
1.4
1.6
1.8
2.0
2.2
2.4
0
0.5
3
6
12
24
48
72
96
Time (hours PBM)

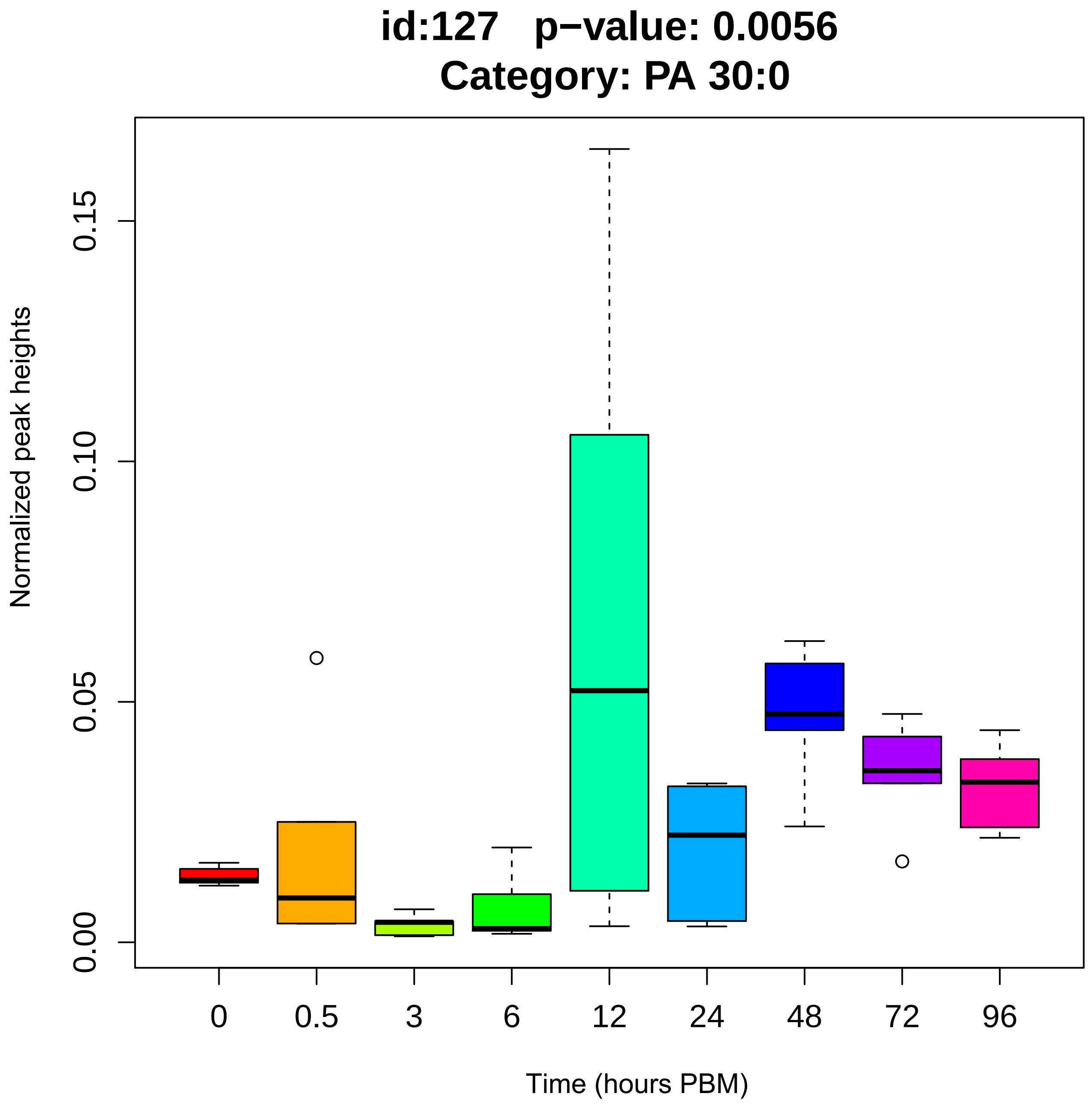

id:127 p−value: 0.0056
Category: PA 30:0
Normalized peak heights
0.00
0.05
0.10
0.15
0
0.5
3
6
12
24
48
72
96
Time (hours PBM)

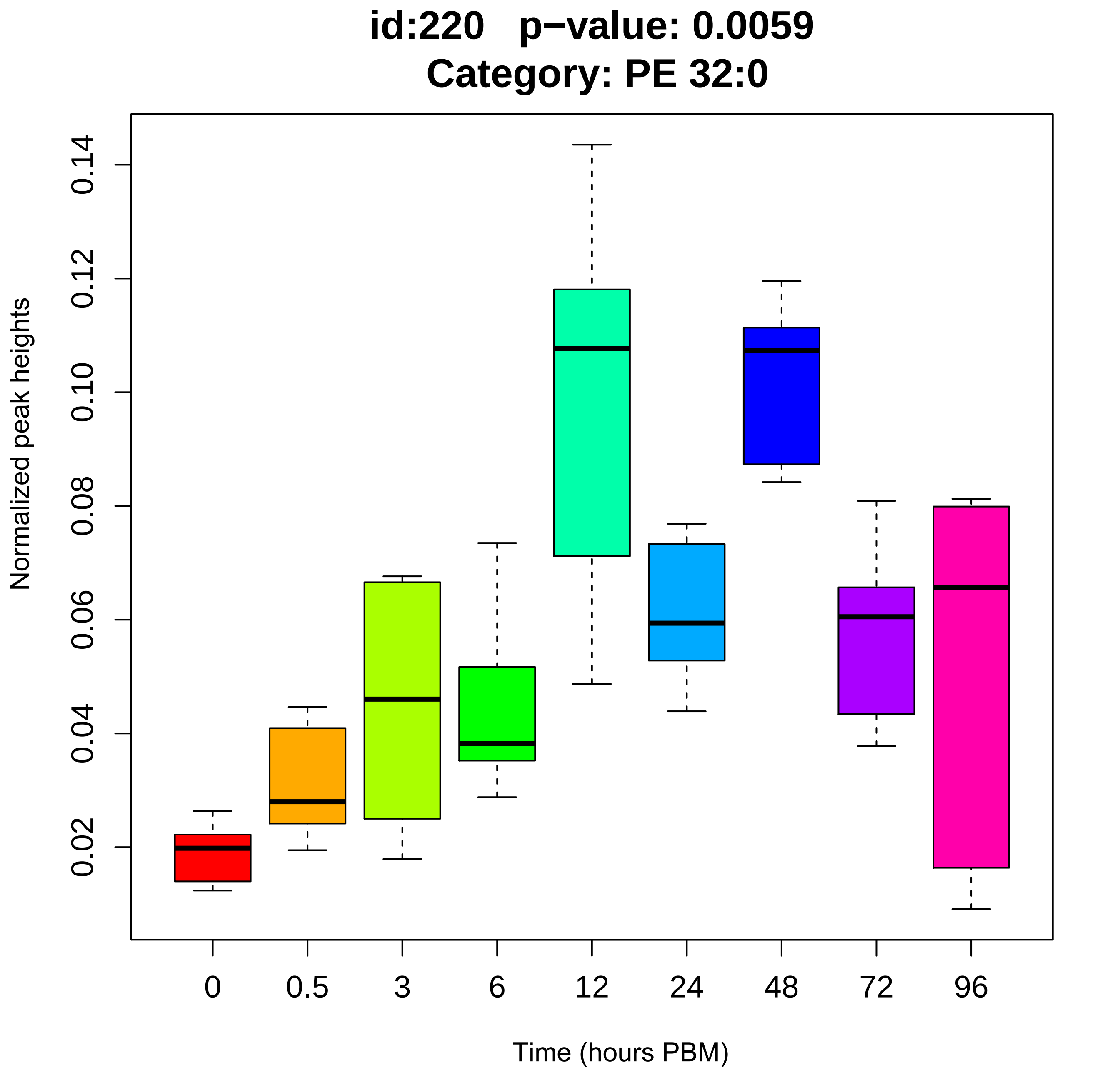
id:220 p−value: 0.0059
Category: PE 32:0
Normalized peak heights
0.02
0.04
0.06
0.08
0.10
0.12
0.14
0
0.5
3
6
12
24
48
72
96
Time (hours PBM)

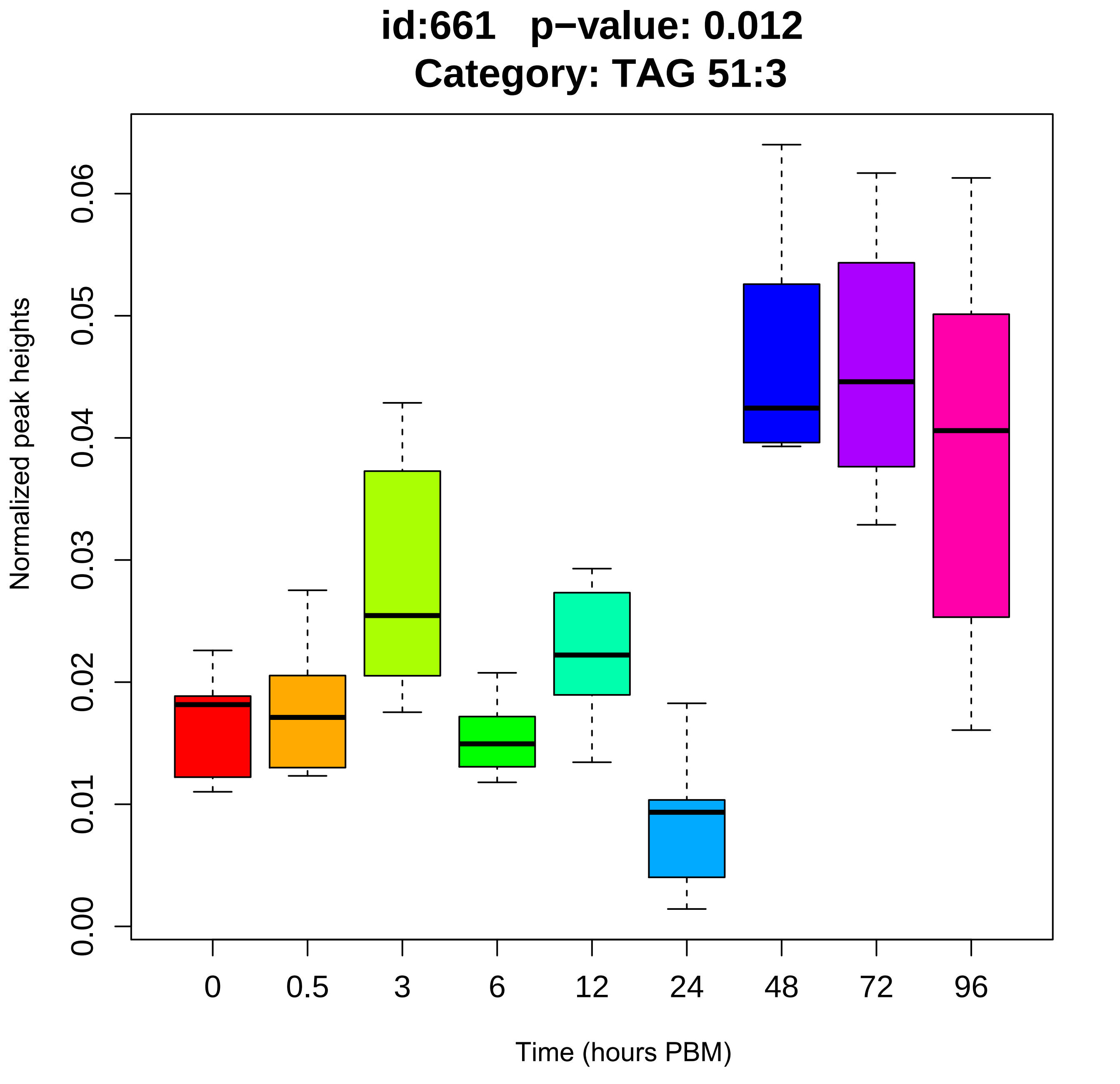
id:661 p−value: 0.012
Category: TAG 51:3
Normalized peak heights
0.00
0.01
0.02
0.03
0.04
0.05
0.06
0
0.5
3
6
12
24
48
72
96
Time (hours PBM)

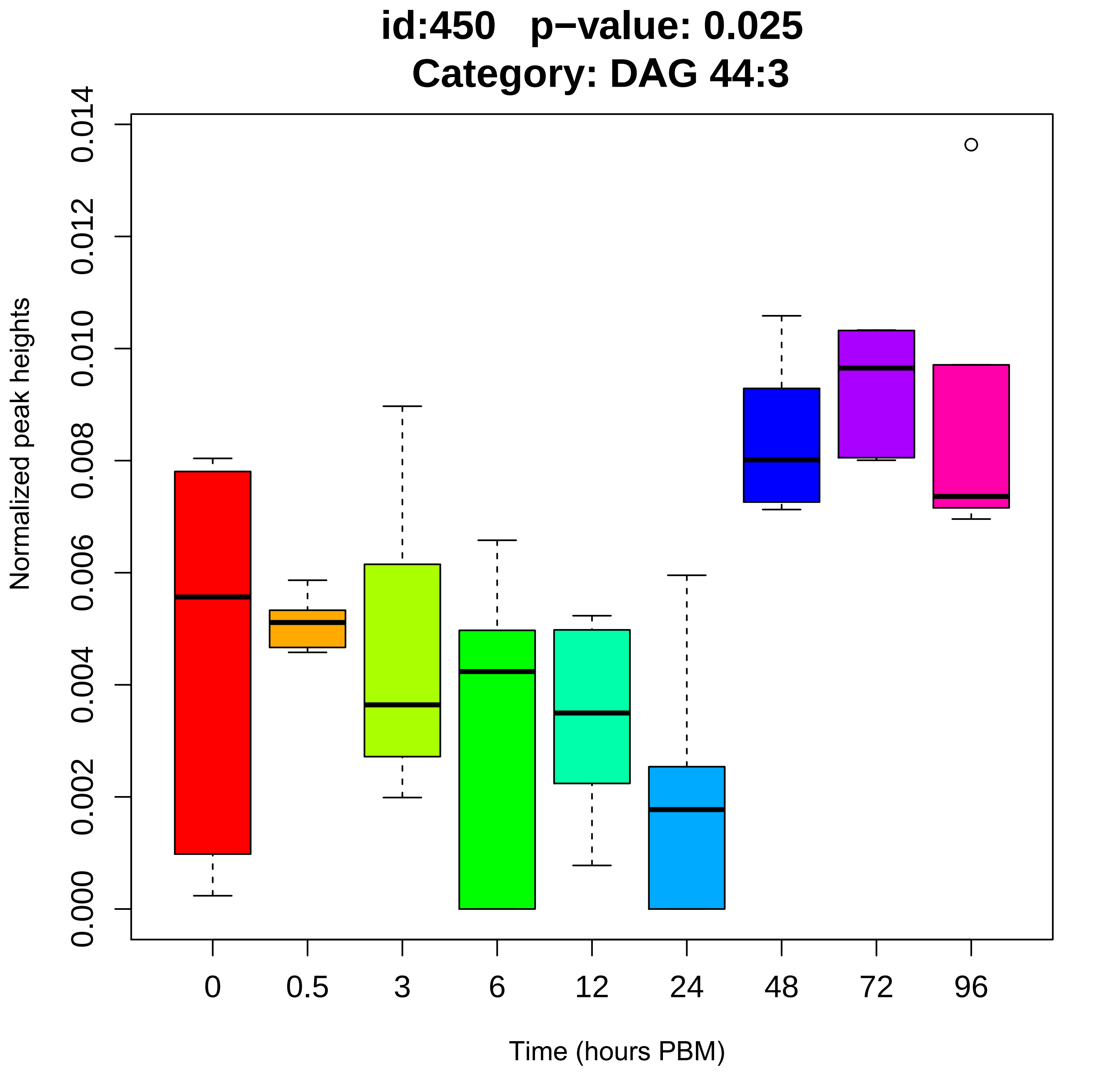
id:450   p−value: 0.025
Category: DAG 44:3
Normalized peak heights
0.000
0.002
0.004
0.006
0.008
0.010
0.012
0.014
0
0.5
3
6
12
24
48
72
96
Time (hours PBM)

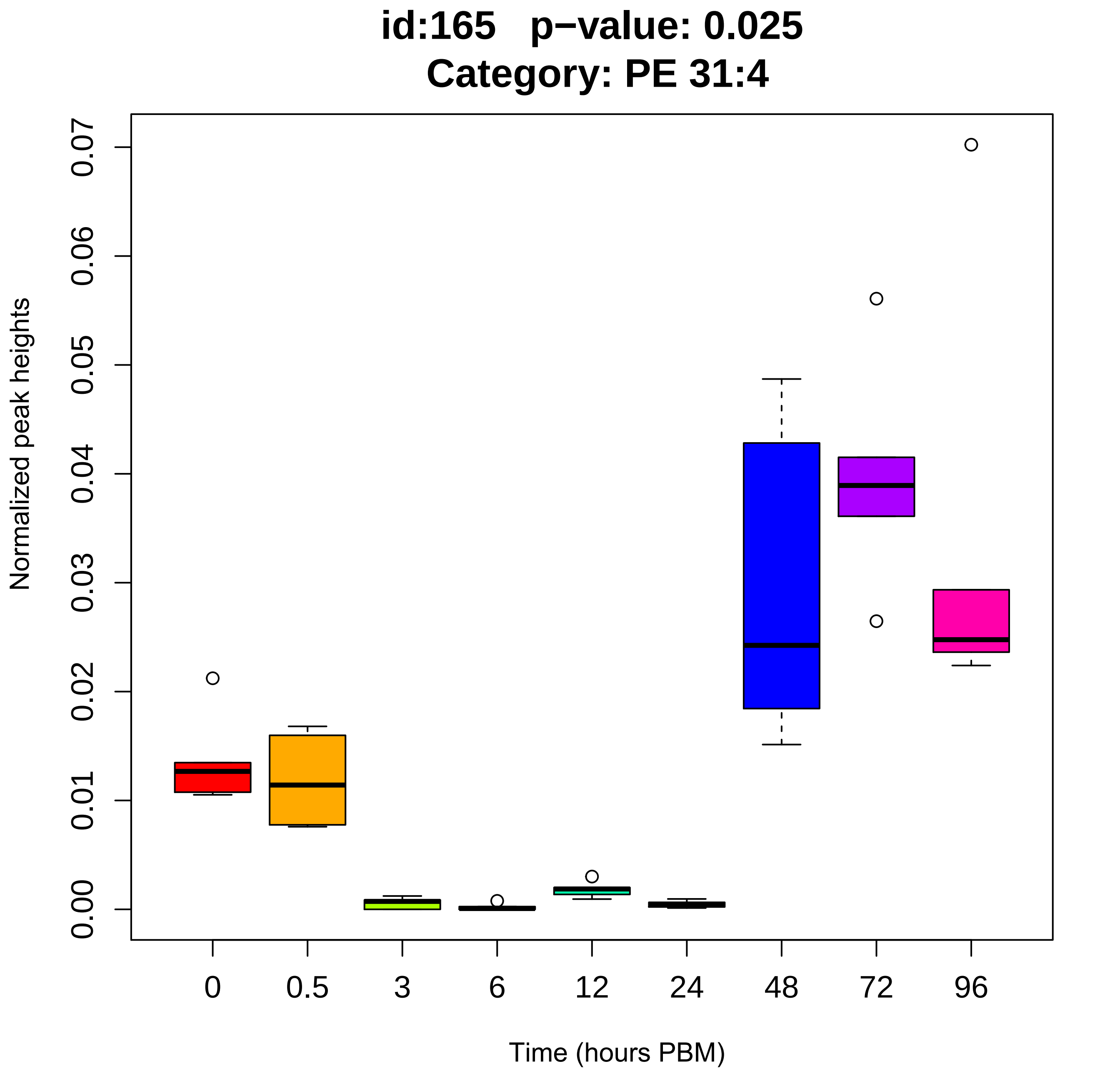

id:165 p−value: 0.025
Category: PE 31:4
Normalized peak heights
0.00
0.01
0.02
0.03
0.04
0.05
0.06
0.07
0
0.5
3
6
12
24
48
72
96
Time (hours PBM)

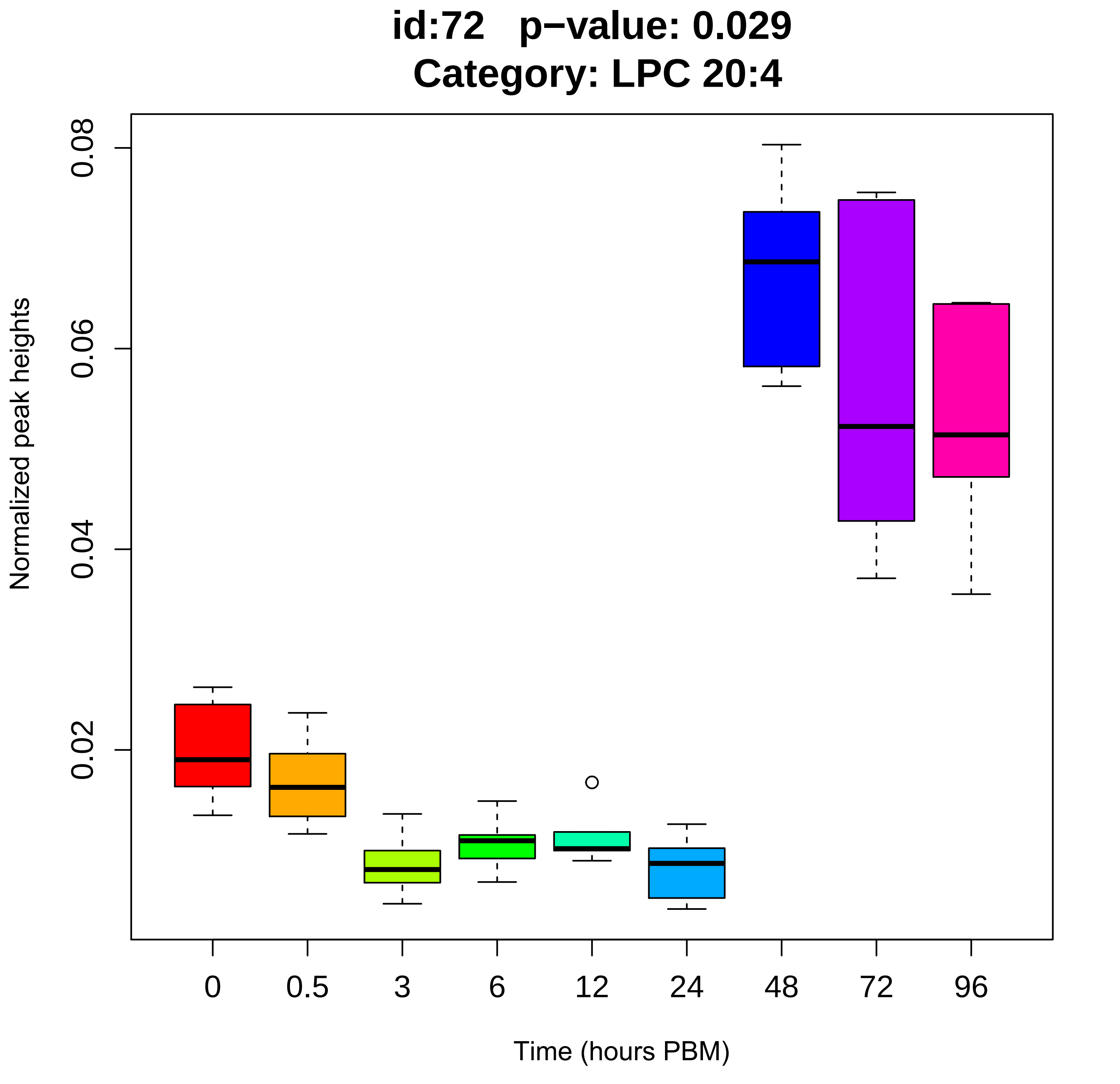

id:72 p−value: 0.029
Category: LPC 20:4
Normalized peak heights
0.02
0.04
0.06
0.08
0
0.5
3
6
12
24
48
72
96
Time (hours PBM)

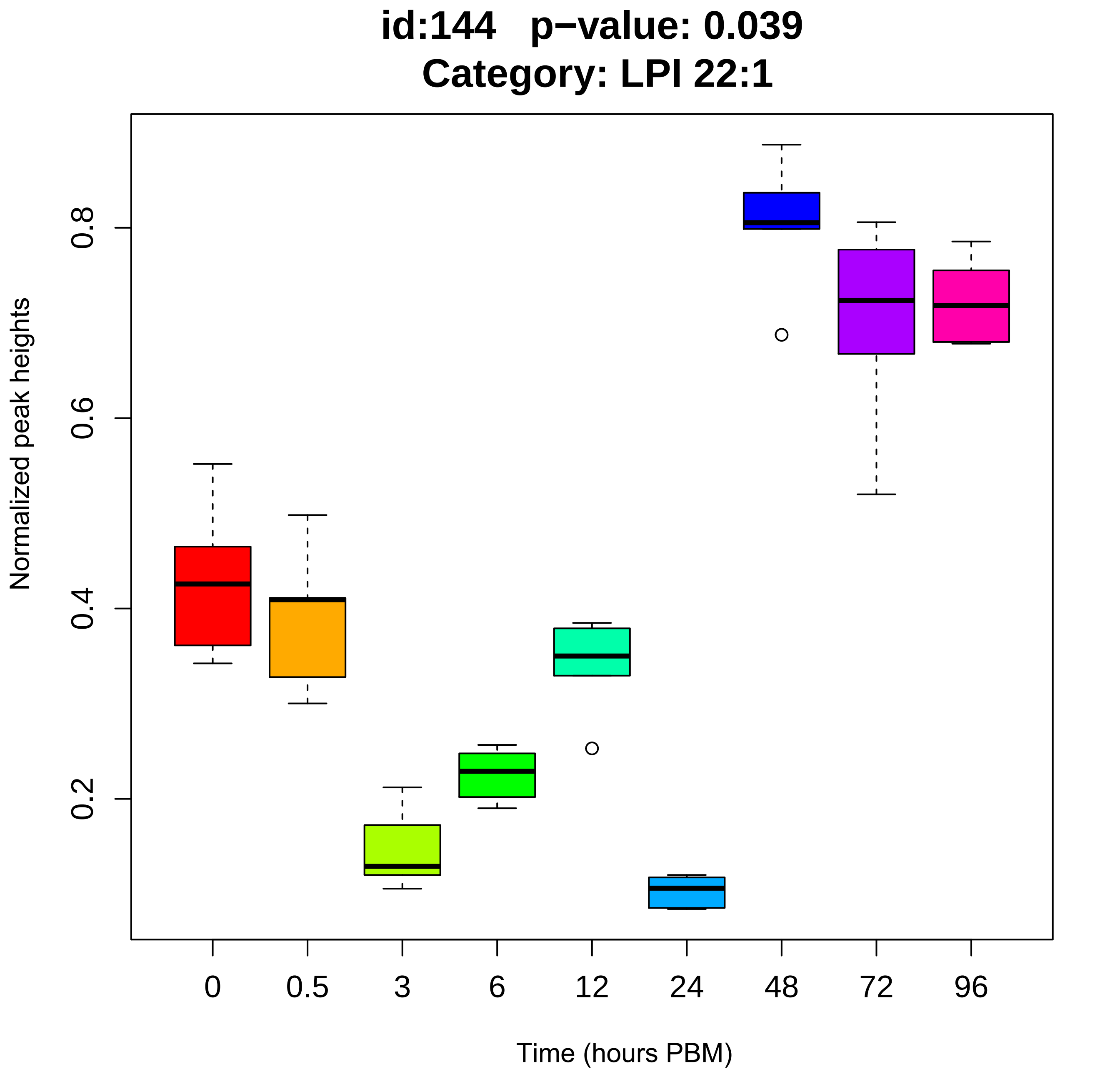

id:144 p−value: 0.039
Category: LPI 22:1
Normalized peak heights
0.2
0.4
0.6
0.8
0
0.5
3
6
12
24
48
72
96
Time (hours PBM)

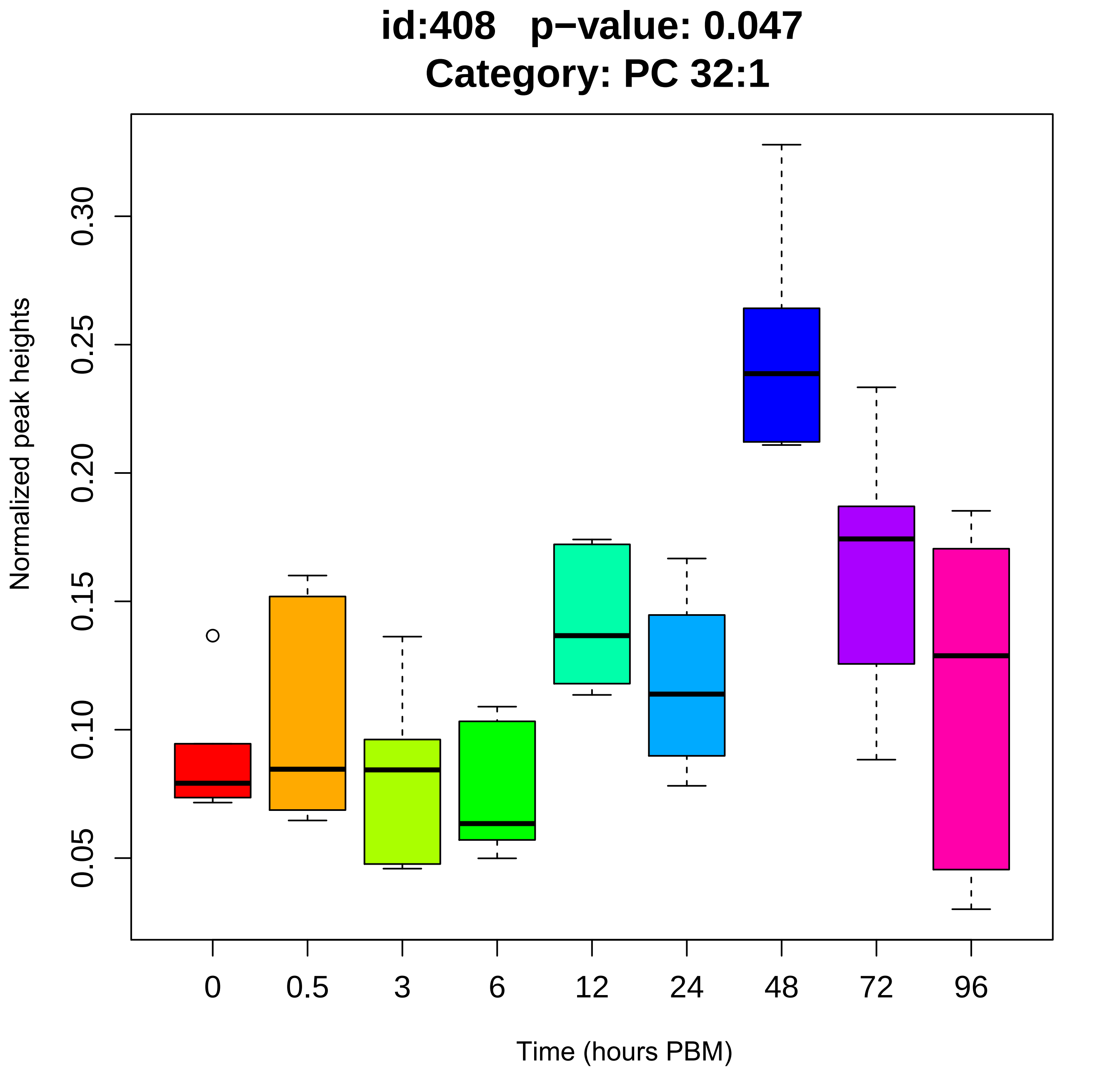

id:408 p−value: 0.047
Category: PC 32:1
Normalized peak heights
0.05
0.10
0.15
0.20
0.25
0.30
0
0.5
3
6
12
24
48
72
96
Time (hours PBM)

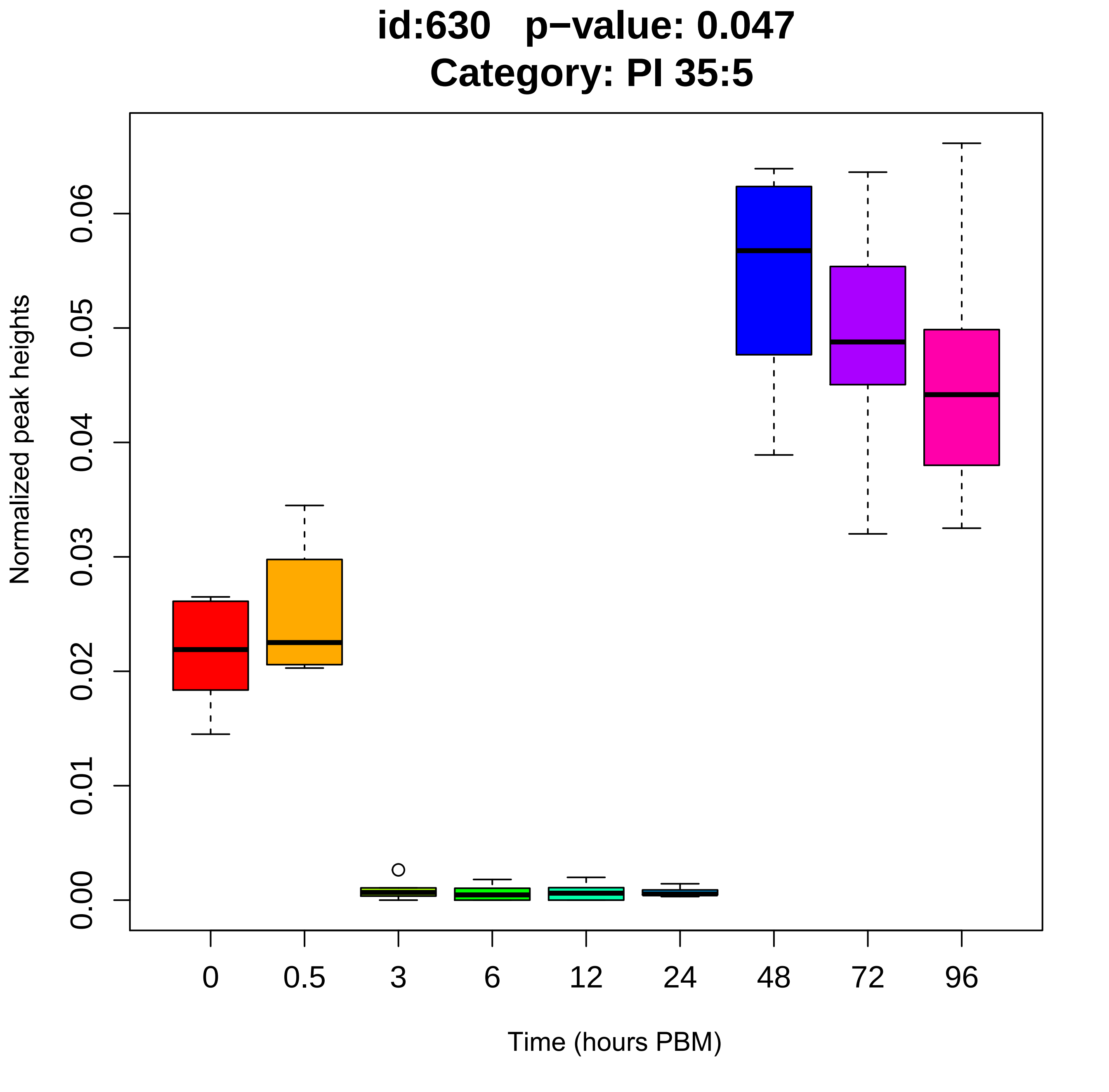

id:630 p−value: 0.047
Category: PI 35:5
Normalized peak heights
0.00
0.01
0.02
0.03
0.04
0.05
0.06
0
0.5
3
6
12
24
48
72
96
Time (hours PBM)
