# Supplementary material for: Fat and Happy: Profiling Mosquito Fat Body Lipid Storage and Composition Post-blood Meal
Source: Front Insect Sci. 2021 Jun 16;1:693168. doi: 10.3389/finsc.2021.693168 (PMC10926494; doi:10.3389/finsc.2021.693168)
Supplement: Supplementary file 5 [file Data_Sheet_4.PDF]

Supplemental File 5. Bar graph illustrating changes in total lipid content over time measured by LC/MS. Letters above bars represent statistically significant differences as determined by Kruskal-Wallis tests ($p<0.05$). Note, the y-axis represents the sum of normalized peak heights for all lipids in the lipidome dataset at each time point. 0 hr PBM time point represents unfed mosquitoes.

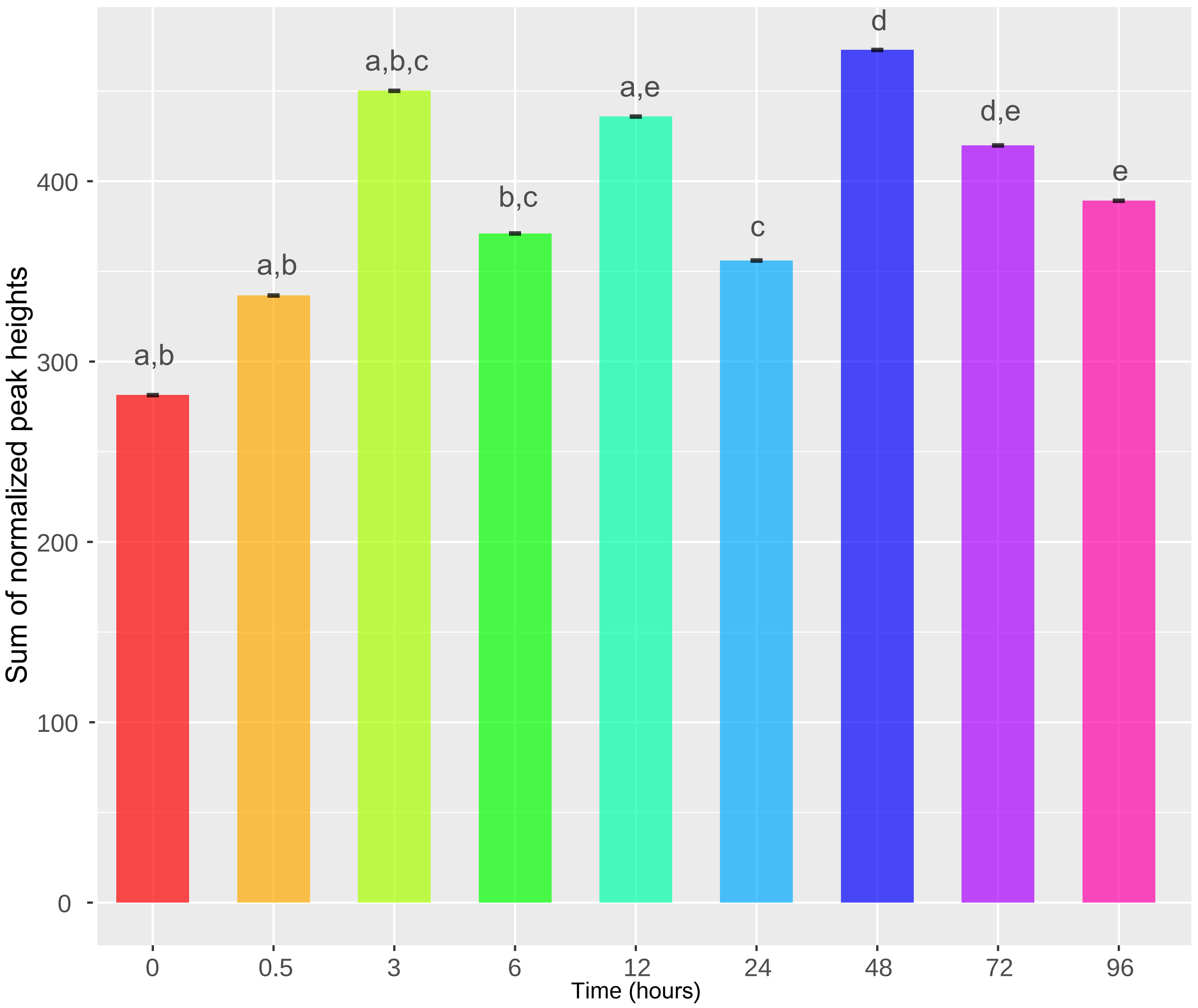

Sum of normalized peak heights
0
100
200
300
400
a,b
a,b
a,b,c
b,c
a,e
c
d
d,e
e
0
0.5
3
6
12
24
48
72
96
Time (hours)
